# Optimizing Electrocoagulation for Textile Effluent Treatment: Operational Efficiency and Environmental Assessment of Remazol Red Dye Removal

**DOI:** 10.1021/acsomega.5c08815

**Published:** 2025-12-12

**Authors:** Francisco F. S. Cruz, Mauro C. C. Góes, Claudemir G. Santana, Tiago G. Santos, Mauricio Boscolo, Rita C. S. Luz, Cícero W. B. Bezerra

**Affiliations:** a Departamento de Química, 37892 Universidade Federal do Maranhão, Av. dos Portugueses, 1966, Bacanga, São Luís, MA CEP 65080-805, Brazil; b Unidade Regional de Educação de São Luís, Secretaria de Educação do Estado do Maranhão, Rua do Cema, 39 - Vila Palmeira, São Luís, MA CEP 65047-400, Brazil; c Departamento de Tecnologia Química, 37892 Universidade Federal do Maranhão, Av. dos Portugueses, 1966, Bacanga, São Luís, MA CEP 65080-805, Brazil; d Instituto de Biociências, Letras e Ciências Exatas − IBILCE/UNESP Campus de São Jose do Rio Preto − SP, Brasil, São Jose do Rio Preto, CEP 15054-000, Brazil

## Abstract

The discharge of textile industrial effluents is a major environmental concern due to their high volume and complex composition. Electrocoagulation (EC) has emerged as a cost-effective, energy-efficient treatment, especially when powered by clean energy. This study optimized standalone EC and its combination with dissolved air flotation (DAF) for treating synthetic effluents containing Remazol Red dye. Fractional factorial and central composite rotational designs were used to optimize potential difference, number and spacing of aluminum electrode pairs, treatment time, initial pH, and dye concentration. EC achieved >98% dye removal under initial conditions and >95% under the optimized model, which showed strong predictive accuracy. No dye was detected in treated samples by HPLC even at high concentrations, and complete removal occurred at lower levels. Energy consumption averaged 4.70 kWh·m^–3^. Sludge generated under optimal conditions was characterized by XRD, FTIR, TGA, and AAS, indicating aluminum oxyhydroxide formation, with potential conversion to aluminum oxide above 293 °C. Results support EC as a viable, sustainable strategy for textile wastewater treatment and resource recovery.

## Graphical abstract

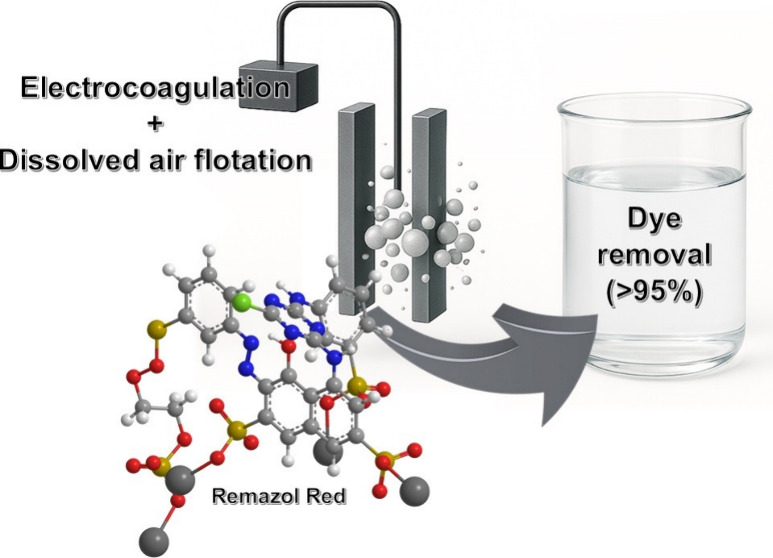

## Introduction

The increase in industrial activity and urbanization has significantly elevated pollutant levels in water bodies.^1–4^ The proliferation of over 100,000 commercial chemicals has made their release into the environment a major concern, as poor management or improper disposal can contaminate aquifers, rendering them unsuitable and environmentally hazardous.^5,6^

These pollutants from industrial effluents are generally classified into two main categories based on the nature of the agents: trace metals and organic compounds.^7^ While some trace metals are essential in low concentrations, they not only can threaten human health and ecosystem stability when elevated but also impact living organisms. Organic pollutants, in turn, are particularly toxic, persistent, and prone to bioaccumulation and biomagnification.^8–10^

The textile industry is a major source of organic contaminants, especially dyes. These compounds reduce light penetration, hinder photosynthesis, are heat-resistant, poorly degradable, and many are carcinogenic.^1,11,12^ Among them, Remazol Red (RR) stands out for its high molar absorptivity, light stability, solubility, and applicability to various fibers. Chemically, it is a reactive azo dye containing sulfonate groups, which confer high solubility and thermal stability, while its conjugated azo bonds impart high resistance to biodegradation. This structural robustness explains its persistence in aquatic environments and the difficulty of its conventional removal. Various treatment approaches, such as adsorption, photocatalysis, and oxidative degradation, have been investigated for RR removal; however, they often require costly reagents or generate secondary byproducts. Its widespread use demands cost-effective, efficient treatment methods to enable reuse and environmental protection.^13,14^

Several remediation strategies, such as adsorption on activated carbon, advanced oxidation processes (AOPs), and photocatalytic degradation, have been extensively explored for dye removal.^15–18^ While these techniques can achieve high removal efficiencies, they often involve high chemical or energy demand and complex operational control, with the added risk of secondary pollution. In contrast, electrocoagulation (EC) integrates coagulant generation and pollutant destabilization into a single step. This process offers significant advantages, including lower sludge production, ease of operation, and cost-effectiveness, thereby making it a particularly attractive option for industrial wastewater treatment.^1,19–22^

Given these advantages, electrocoagulation (EC) has emerged as a promising and widely studied technique for the treatment of industrial effluents. This electrochemical method uses sacrificial electrodes to generate coagulant species *in situ* destabilizing pollutants through charge neutralization and aggregation. EC is simple, efficient, reliable, and cost-effective, with low sludge generation and minimal chemical additives.^23^

At the anode, the electrode is oxidized:

$$M_{\left(s\right)}\to M_{\left(\mathrm{aq}\right)}^{n+}+{\mathrm{ne}}^{-}$$ 1

At the cathode, water is reduced:

$$2H_{2}O_{\left(l\right)}+2e^{-}\to 2{\mathrm{OH}}_{\left(\mathrm{aq}\right)}^{-}+H_{2\left(g\right)}$$ 2

Although water oxidation is thermodynamically favored (E^◦^ = + 1.23V, eq 3), iron (E^◦^ = + 0.44/+0.77 V) and aluminum (E^◦^ = + 1.66 V) electrodes are preferentially oxidized due to faster kinetics and lower activation energy. Passive oxide films (e.g., Al_2_O_3_) can sometimes promote water oxidation by hindering metal dissolution.

$$2H_{2}O_{\left(l\right)}\to 2O_{2\left(g\right)}+4H_{\left(\mathrm{aq}\right)}^{+}+4e^{-}$$ 3

EC efficiency depends on factors such as electrode material, current density, voltage, pH, temperature, conductivity, pollutant concentration, electrolysis time, gas bubble accumulation, electrode spacing, and mass transfer.^1^ Reactor design and electrode arrangement also influence performance. Hydrogen bubbles from water reduction may aid flotation but can also increase resistance by forming insulating layers.^1,24^ Given this complexity, experimental design emerges as an essential tool for optimizing EC, enabling identification of significant variables, quantification of their effects and interactions, and the development of predictive mathematical models.^24,25^

Unlike many previous EC studies that were primarily limited to operational optimization, the present work integrates factorial and central composite designs to develop predictive models, while simultaneously addressing critical practical aspects such as electrode consumption, corrosion behavior, sludge characteristics, and regulatory compliance. This holistic approach provides a more comprehensive assessment of the feasibility and sustainability of EC-based treatments.^21,22^

EC is also attractive for integration with other treatment methods. One example is dissolved air flotation (DAF), which uses microbubbles and is commonly applied in mining, drinking water, and wastewater treatment.^21,26–28^ EC may also be enhanced with auxiliary flocculants.^22^ Although energy intensive, EC energy demand can be reduced by optimizing conductivity and electrode spacing, and by employing clean energy sources such as photovoltaics.^29–32^

This study aimed to determine optimal conditions for treating RR dye solutions using combined EC-DAF. A two-step experimental design was used: a fractional factorial design (2^k‑p^) to screen significant variables and a central composite rotatable design (CCRD) for fine-tuning and modeling. Six operational variables were considered: potential difference (PD), electrode pairs (EP), electrode spacing (ES), electrolysis time (t), initial pH (pH_i_), and dye concentration (C). Experiments were conducted in batch mode with aluminum electrodes. Performance was evaluated by monitoring salinity (Sal), conductivity (Cond, Λ), total dissolved solids (TDS), final pH (pH_f_), color (Col), turbidity (Tur), and removal rate (Rr).

## Experimental Section

### Chemicals

Analytical grade chemicals were used throughout the experiment, including sodium chloride (NaCl, ISOFAR), hydrochloric acid (HCl, QUIMEX), sodium hydroxide (NaOH, QUIMEX), potassium bromide (KBr, ISOFAR), nitrogen gas (N_2_, White Martins), ethanol (C_2_H_6_O, QUIMEX), aluminum nitrate (Al­(NO_3_)_3_, ISOFAR). Remazol Red dye (DyStar) was provided by Toalhas São Carlos Industry (São Paulo, Brazil). The aluminum was purchased from a local store, and the electrodes were rolled to dimensions of 10 cm × 5 cm x 1 mm thick. Distilled water was used to prepare all the solutions and the synthetic effluent.

### Preparation and Characterization of the Synthetic Effluent

The synthetic effluent was prepared by dissolving Remazol Red dye in a 1.0 gL^–1^ NaCl solution to impart an ionic nature to the solution. The solution was characterized before and after each treatment by measuring color according to method 2021 D from Standard Methods,^33^ turbidity, and conductivity. Table 1 shows the initial characteristics of the Remazol Red solutions prepared for the electrocoagulation tests. Calibration curves for the dye were constructed at various pH levels (2 to 12) and in the same ionic medium (1.0 gL^–1^ NaCl). Hydrochloric acid or sodium hydroxide was used to adjust the pH.

**Table 1:** Characteristics of Remazol Red Solutions: Average Values (± Standard Deviation) for Conductivity and Turbidity Parameters as a Function of Initial pH^a^

pH	C_i_ (mg L^–1^)	Λ (mS cm^–1^)	Tur (NTU)
5.0	10	2.04 ± 0.02	0.19 ± 0.08
	150	2.37 ± 0.30	1.99 ± 0.38
8.5	80	1.92 ± 0.30	0.72 ± 0.08
12.0	10	2.19 ± 0.43	0.21 ± 0.11
	150	2.59 ± 0.44	1.39 ± 0.29

a C_i_: initial concentration; Λ: conductivity; Tur: turbidity.

### Instrumentation and Experimental Conditions

The dependent variables were monitored using the following equipment: a Quimis Q400AS pH meter, an ITTB100 turbidimeter, a Quimis Q400AS conductivity meter, and a Kasuaki IL-592S UV–visible spectrophotometer. A Hikari HF-3203S power supply was used in the electrocoagulation (EC) processes, and the Mega Air CFA 7.6/24L compressor was used in the pressure tests with dissolved air.

The Remazol Red dye, widely used industrially and potentially containing unspecified additives, was analyzed using high-performance liquid chromatography (HPLC), thermogravimetric analysis (TGA), Fourier-transform infrared spectroscopy (FTIR), X-ray diffraction (XRD), and UV–vis spectrophotometry. The Supporting Information (SM) provides detailed results from these analyses.

The EC sludge was characterized using AAS, XRD, TGA, and FTIR to better understand its nature and contribute to developing protocols for its reuse or disposal. Prior to characterization, it was predried in an oven at 100 °C for 24 h. A comprehensive data set is included in the SM.

The aluminum electrodes were characterized before and after use through AAS, XRD, and scanning electron microscopy with energy-dispersive X-ray spectroscopy (SEM-EDX). The findings from these analyses are discussed in detail in the SM.

HPLC measurements were conducted with a Shimadzu NexeraXR system equipped with a UV–vis diode array detector (SPD-M20A) set to monitor wavelengths from 200 to 800 nm. The mobile phase consisted of water and methanol, and separation was achieved using a C18 Supelco column. An isocratic elution (50:50%) was employed for 25 min at a flow rate of 0.5 mL min^–1^. The injection volume was 20 μL, with the column temperature maintained at 22 °C, the injector temperature at 25 °C, and the analysis cell temperature also at 25 °C.

TGA analyses were performed using a PerkinElmer TGA 4000 instrument. The samples were analyzed in a synthetic air atmosphere. The thermal program included an initial hold at 50 °C for 1 min, followed by a heating ramp from 50 to 800 °C at a rate of 20 °Cmin^–1^, and a final hold at 800 °C for 5 min.

Infrared Spectroscopy measurements were recorded on a PerkinElmer UART. The spectral range was set from 4000 to 400 cm^–1^, and four spectra were accumulated for each sample. Samples were directly placed onto the diamond ATR (Attenuated Total Reflectance) crystal.

XRD patterns were obtained using a Rigaku MiniFlex300 diffractometer. The instrument utilized CuKα radiation (λ = 0.154 nm) and operated at 40 kV and 30 mA. The 2θ range was scanned from 5 to 80°. Samples were placed directly onto a quartz sample holder.

A Varian AA240FS Spectrometer was used with a nitrous oxide—acetylene flame (11.00 Lmin^–1^ and 6.95 Lmin^–1^, respectively) to determine aluminum in the electrodes, sludge, and treated effluent. The electrode and sludge materials were previously solubilized in concentrated hydrochloric acid. Calibration curves were prepared using aluminum nitrate standards at concentrations of 10, 20, 30, 50, and 80 mgL^–1^.

The morphology and elemental composition of the aluminum electrodes were analyzed using a Zeiss EVO-LS15 scanning electron microscope (SEM) equipped with a secondary electron detector and an Oxford Instruments Inca energy-dispersive X-ray spectroscopy (EDX) detector featuring a resolution of 133 eV. The samples were prepared by sputter-coating with a thin layer of gold to enhance conductivity. SEM images were acquired at an accelerating voltage of 20 kV, and EDX analysis was performed simultaneously to determine the elemental composition.

### Electrochemical Reactor and Experimental Configuration

The electrochemical reactor used in the experimental tests consisted of an acrylic tank with the following dimensions: 3.0 mm thickness, 30 cm length, 15 cm width, and 25 cm height. It had a removable lid for fitting aluminum electrodes with a surface area of 5.0 cm^2^ each. The slots for electrode insertion were spaced 1.0 cm apart, totaling 22 openings. A cylindrical device, 1.0 cm in diameter and 30 cm in length, was installed at the bottom of the reactor. This device had small holes throughout its surface to ensure better distribution of dissolved air, functioning as a diffuser in the dissolved air flotation (DAF) chamber. An adjustable power supply powered the system.

The dissolved air production chamber was made of 5 mm thick stainless steel, with external dimensions of 16 cm in diameter and 25 cm in height. The top of the cylinder was equipped with a pressure gauge and two valves: one for water inlet and the other for air inlet from a compressor. The air inlet extended internally to the bottom of the cylinder for better water saturation. An external level gauge was added to the upper side of the chamber to control the water volume inside the cylinder. Figure 1 shows the experimental setup used for the EC-DAF tests.

**Figure 1:**
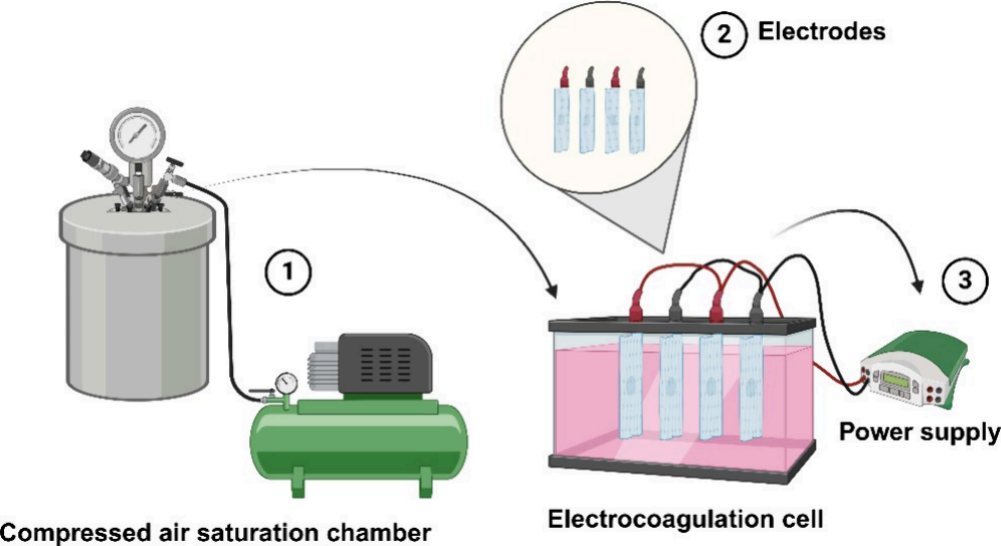
Experimental setup for EC-DAF in the treatment of remazol red synthetic effluent.

All EC experiments were conducted at 25 °C. Each test used 3 L of dye solution at 10, 80, and 150 mg L^–1^ concentrations in a 1.0 g L^–1^ NaCl ionic medium. The initial pH of the solutions was adjusted with HCl or NaOH as needed to ensure the system’s initial condition. The electrodes were washed with distilled water and ethanol and dried in an oven at 100 °C for 10 min before each test. The electrodes were then inserted into the cell and connected to the DC power supply. Atmospheric air was dissolved in water using the compressor until the pressure inside the chamber reached 4 atm.

The power supply was turned off at the end of the electrolytic process. Air/water from the saturation chamber was injected into the bottom of the reactor through the diffuser tube. After 15 min of dissolved air injection under pressure, a 100 mL sample of the treated solution was collected from an outlet at the bottom of the cell, filtered using qualitative filter paper, and analyzed for salinity, conductivity, total dissolved solids, final pH, color, turbidity, and dye removal rate. The removal efficiency was calculated using eq 4.

$$\%Rr=\frac{C_{i}-C_{f}}{C_{i}}\times 100$$ 4

Where *C*_*i*_ and *C*_*f*_ are the initial and final dye concentrations (mg L^–1^), respectively. The total dissolved solids and salinity measurements were indirectly calculated from conductivity values using eqs 5 and 6.^34,35^

TDS=0.640Cond 5

$$Sal={Cond}^{1.0878}0.4665$$ 6

Where TDS is the concentration of total dissolved solids (mg L^–1^), *Cond* is the electrical conductivity of the medium (μScm^–1^), and *Sal* is the salinity (g L^–1^).

### Experimental Design

A sequential experimental design was used to optimize the EC-DAF process. Initially, a fractional factorial design (2^k‑p^) was employed to evaluate significant independent variables (*p* < 0.1). Table 2 shows the defined values for each parameter studied and the levels and independent variables.

**Table 2:** Variables, Levels, and Values Used in the Fractional Factorial Design (FFD) for the Dye

**variables/unit**	**levels**		
	–1	0	+1
	**real values**		
PD/V	5	12.5	20
EP/u	1	2	3
ES/cm	1	2	3
t/min	30	75	120
pH_i_	5	8.5	12
C_i_/mg L^–1^	10	80	150

The operational variables selected—potential difference (PD), electrode pairs (EP), electrode spacing (ES), electrolysis time (t), initial pH (pHi), and dye concentration (C)—were chosen due to their direct influence on the efficiency, kinetics, and stability of the electrocoagulation process. The applied PD controls the current density and the rate of coagulant generation, while EP (number of electrode pairs) determines the total effective surface area available for electrochemical reactions, influencing current distribution and treatment capacity. ES affects the electrical resistance of the system and the hydrodynamic conditions for floc formation. Electrolysis time (t) governs the duration of coagulant production and pollutant contact. The initial pH (pHi) regulates the speciation of metal hydroxides and dye ionization, thereby modulating coagulation and adsorption efficiency. Finally, the initial dye concentration (C) defines the pollutant loading, guiding process optimization toward maximum removal efficiency. Together, these parameters represent the main physicochemical factors controlling EC performance and were systematically varied to ensure comprehensive process optimization and modeling.^12^

Subsequently, a central composite rotational design (CCRD) was used to optimize and determine optimal conditions based on the operating range of the system under study.

The results were evaluated using STATISTICA version 9.0 software. A two-level fractional factorial design (FFD) was used to determine statistically significant operational variables, with 16 factorial trials and 5 central points. The central composite rotational design (CCRD) involved 51 trials, including 12 axial points (α) and seven central points.

Mathematical relationships for fitting error and relative error, as shown in eqs 7 and 8, were used to validate the model. These relationships assess the similarity between the experimental and predicted model responses.^21,36,37^

$$E_{\mathrm{adjustment}}=Y-Ŷ$$ 7

$$E_{\mathrm{relative}}=\frac{E_{\mathrm{adjustment}}}{Y}\times 100$$ 8

Where *Y* corresponds to the experimental value and *Ŷ* to the predicted response by the model.

Alongside the analysis of response variables, the effect of dissolved air pressure on the EC process efficiency was investigated, and temperature and electrode consumption were monitored.

The Tukey test was used to investigate potential differences between treatments, including the pressure variable. This statistical tool compares multiple means to identify pairs with significant differences.^38^ For this purpose, four experimental groups were established under the same conditions (central points), differing only in the presence or absence of dissolved air pressure at the end of each trial. The variable ″removal″ was analyzed to assess possible differences. The categorical groups were FFD without pressure, FFD with pressure, CCRD without pressure, and CCRD with pressure.

### Desirability Function (DF)

This function aims to show the optimal values for each variable analyzed through the CCRD when multiple response variables are involved. The desirability function is based on a numerical range from 0 to 1, where 1 and 0 represent maximum and minimum desirability, respectively, as described in eqs 9–11.^37,39,40^

$$(df_{i})={\left(\frac{U-\alpha }{\beta -\alpha }\right)}^{wi},\alpha \leq U\leq \beta$$ 9

$$\left(df_{i}\right)=1,U>\beta$$ 10

$$\left(df_{i}\right)=1,U<\alpha$$ 11

In eq 9, α and β are the respective minimum and maximum values obtained from the response i, and w_i_ is the level of importance. The individual desirability scores for the predicted values of each dependent variable are combined with the DF function using their geometric means over all the different values of (df_i_).^40,41^

## Results and Discussion

### Fractional Factorial Design (FFD)

Table 3 presents the results of treating synthetic effluent under fractional factorial design (FFD) conditions. Several trials (16–21) demonstrated significant removals, with values above 96%, indicating effective treatment performance.^1^ These trials also showed color and pH values within an acceptable range, further confirming the process’s efficacy. The FFD was instrumental in performing a preliminary and exploratory analysis, which enabled identifying and selecting significant independent variables that influence the electrocoagulation (EC) process.

**Table 3:** Actual Experimental Design Matrix and Response Data for Electrocoagulation (EC) of Remazol Red Effluent, with and without Coupling to DAF: Conductivity, Salinity, TDS, Final pH, Color, Turbidity, and Dye Removal Efficiency^a^

run	PD (V)	EP (unid.)	ES (cm)	T (min.)	pH_i_	C_i_ (mg L^–1^)	Λ (mS cm^–1^)	Sal (g L^–1^)	TDS (ppm)	pH_f_	Col	Tur (UNT)	Rr (%)
19 (c)	12.5	2	2	75	8.50	80	1.78	0.87	1.14	8.50	0.05	0.20	96.8
19 (c)/DAF							0.00	0.00	0.00	8.50	0.10	0.28	95.0
21 (c)							2.92	1.49	1.87	7.85	0.00	0.34	96.9
21 (c)/DAF							1.58	0.77	1.01	8.80	0.00	0.17	97.2
15	5	3	3	120	5.00	150	2.11	1.05	1.35	8.58	0.74	0.22	74.7
15/DAF							2.03	1.01	1.30	8.89	0.74	0.17	69.4
7	5	3	3	30	5.00	10	1.80	0.89	1.15	9.55	0.00	0.25	93.5
7/DAF							1.62	0.79	1.04	9.63	0.00	0.10	98.0
2	20	1	1	30	12.00	10	9.97	5.69	6.38	11.10	0.50	0.13	29.0
2/DAF							2.28	1.14	1.46	10.85	0.50	0.29	37.3
5/SP	5	1	3	30	12.00	150	2.86	1.46	1.83	11.53	0.86	1.94	7.45
5/DAF							2.61	1.32	1.67	11.27	0.90	1.18	8.04
18 (c)	12.5	2	2	75	8.50	80	2.31	1.16	1.48	8.07	0.20	0.37	96.6
18 (c)/DAF							1.66	0.81	1.06	8.95	0.20	0.42	96.5
16	20	3	3	120	12.00	150	2.80	1.43	1.79	8.90	0.06	0.22	98.0
16/DAF.							2.13	1.06	1.36	9.70	0.83	0.08	96.2
8	20	3	3	30	12.00	10	1.64	0.80	1.05	9.26	0.06	0.12	52.0
8/DAF							1.62	0.79	1.04	9.61	0.06	0.42	72.3
12	20	3	1	120	5.00	10	1.66	0.81	1.06	9.16	0.00	0.13	92.0
12/DAF							1.17	0.55	0.75	9.14	0.00	0.01	91.8
1	5	1	1	30	5.00	10	1.63	0.79	1.04	8.51	0.98	0.10	53.0
1/DAF							1.76	0.86	1.13	8.20	0.88	0.10	93.0
17 (c)	12.5	2	2	75	8.50	80	1.12	0.53	0.72	7.65	0.00	0.49	97.4
17 (c)/DAF							1.59	0.77	1.01	8.63	0.00	0.45	97.3
9	5	1	1	120	5.00	150	2.33	1.17	1.49	8.75	0.74	0.42	65.2
9/DAF							1.78	0.87	1.14	8.67	0.80	0.46	77.2
4	20	3	1	30	5.00	150	2.03	1.01	1.30	8.30	0.77	0.18	91.4
4/DAF							1.66	0.81	1.06	8.50	0.71	0.10	61.6
13	5	1	3	120	12.00	10	2.03	1.01	1.30	10.88	0.09	0.44	0.80
13/DAF							1.74	0.85	1.11	10.22	0.11	0.13	0.21
20 (c)	12.5	2	2	75	8.50	80	2.68	1.36	1.72	9.13	0.12	0.26	95.6
20 (c)/DAF							1.78	0.88	1.14	9.43	0.05	0.10	96.4
3	5	3	1	30	12.00	150	1.97	0.97	1.26	9.40	0.75	0.31	22.3
3/DAF							1.52	0.74	0.97	9.10	0.75	0.61	72.7
10	20	1	1	120	12.00	150	2.48	1.25	1.59	9.90	0.50	0.45	74.4
10/DAF							2.31	1.16	1.48	9.76	0.40	0.27	79.8
11	5	3	1	120	12.00	10	1.86	0.92	1.19	10.94	0.67	0.56	8.70
11/DAF							1.18	0.56	0.76	11.06	0.80	0.16	5.89
14	20	1	3	120	5.00	10	1.94	0.96	1.24	7.60	0.50	0.92	81.0
14/DAF							1.53	0.74	0.98	7.75	0.00	0.62	92.0
6	20	1	3	30	5.00	150	2.11	1.05	1.35	9.05	0.93	1.23	38.4
6/DAF							1.77	0.87	1.13	8.60	0.93	0.20	57.5

a The symbol (c) denotes the central point of EC conditions defined by the factorial design. PD: Potential difference, EP: electrode pairs, ES: electrode space, T: time, pH_i_: initial pH, C_i_: initial concentration, conductivity: Λ, Sal: salinity, TDS: total dissolved solids, pH_f_: final pH, Col: color, Tur: turbidity, Rr: removal rate.

Dissolved air pressure (DAF) influence was examined across all FFD trials. Literature indicates that the efficiency of the EC process is closely related to the size, intensity, and distribution of the generated bubbles. Smaller and better-distributed bubbles within the system result in more efficient treatment.^42–44^ The advantages of coupling EC with DAF typically include reducing the retention time of the treated effluent, lowering electrode consumption, and providing better responses for weakly coagulated systems.^42–44^ For this study, DAF conditions were standardized for all trials, as shown in Table 3.

The effect of dissolved air pressure (DAF) on the removal efficiency of Remazol Red was statistically evaluated using ANOVA and Tukey’s test. Four experimental groups were analyzed under central point conditions: FFD without pressure, FFD with pressure, CCRD without pressure, and CCRD with pressure. The results indicated no statistically significant differences between the removal efficiencies with and without applying pressure (p-value > 0.05, Fcal < Fcritical). The removal rates ranged from 94.5 to 97.4% without pressure and from 89.5 to 97.3% with pressure, demonstrating that adding pressure did not enhance the removal process under the tested conditions.

Although the coupling of EC with DAF is widely reported to yield synergistic benefits in terms of sludge separation and energy consumption,^22,44,45^ our statistical results indicate that, under the standardized operating conditions, the DAF unit did not provide a statistically significant synergistic effect on the overall removal efficiency. This outcome can be attributed to the high effectiveness of the EC-only process, which was already operating at or near its maximum removal capacity for the Remazol Red dye.

The DAF process involves significantly greater complexity than merely introducing pressurized air. Its efficiency depends on a delicate balance of three-phase fluid dynamics, where successful bubble–particle interactions (collision, adhesion, and drag forces) are highly sensitive to both external and operational variables. The required air volume for effective flotation is determined by the concentration and size of suspended particles, while the loading capacity depends on the total bubble surface area. This balance is influenced by numerous design and environmental parameters, including hydraulic loading capacity (HLC), surface loading rate (SLR), recirculation rate (%R), gas holdup, temperature, and salinity. Salinity, for instance, has been shown to affect air dissolution, bubble characteristics, and system hydrodynamics.^44–46^

Given that the DAF conditions in this study were standardized (fixed saturator pressure and hydraulic retention time) and not independently optimized for the comprehensive set of physical and chemical variables—particularly those required to match the specific density and morphology of flocs generated during aluminum-based EC of Remazol Red—the absence of statistically significant improvement is attributed to a suboptimal integration between the processes. The standardized DAF parameters likely failed to generate sufficient lift force or the optimal bubble-to-floc surface area ratio needed to enhance separation beyond the already efficient natural electroflotation occurring within the EC unit.

In light of these results, including DAF was deemed unnecessary for subsequent experiments. This decision simplifies the experimental setup and reduces operational complexity while maintaining the high efficiency already achieved without pressurization. The SMs provide details of the statistical calculations, along with the corresponding tables (Tables S1 to S3) and Figure (S1), for reference.

The Pareto charts for the FFD (Figure 2) accurately illustrate the statistically significant parameters by response variables. Those whose values exceed the dotted line, defined at *p* < 0.1, should be included in the central composite rotational design (CCRD) and possibly considered in the mathematical model. The factors in the charts are ordered from top to bottom, allowing the most significant ones to be identified. Positive values indicate that the response increases with the increase in the working range for each variable. Similarly, when the sign is negative, the response decreases with the increase in the variable.

**Figure 2:**
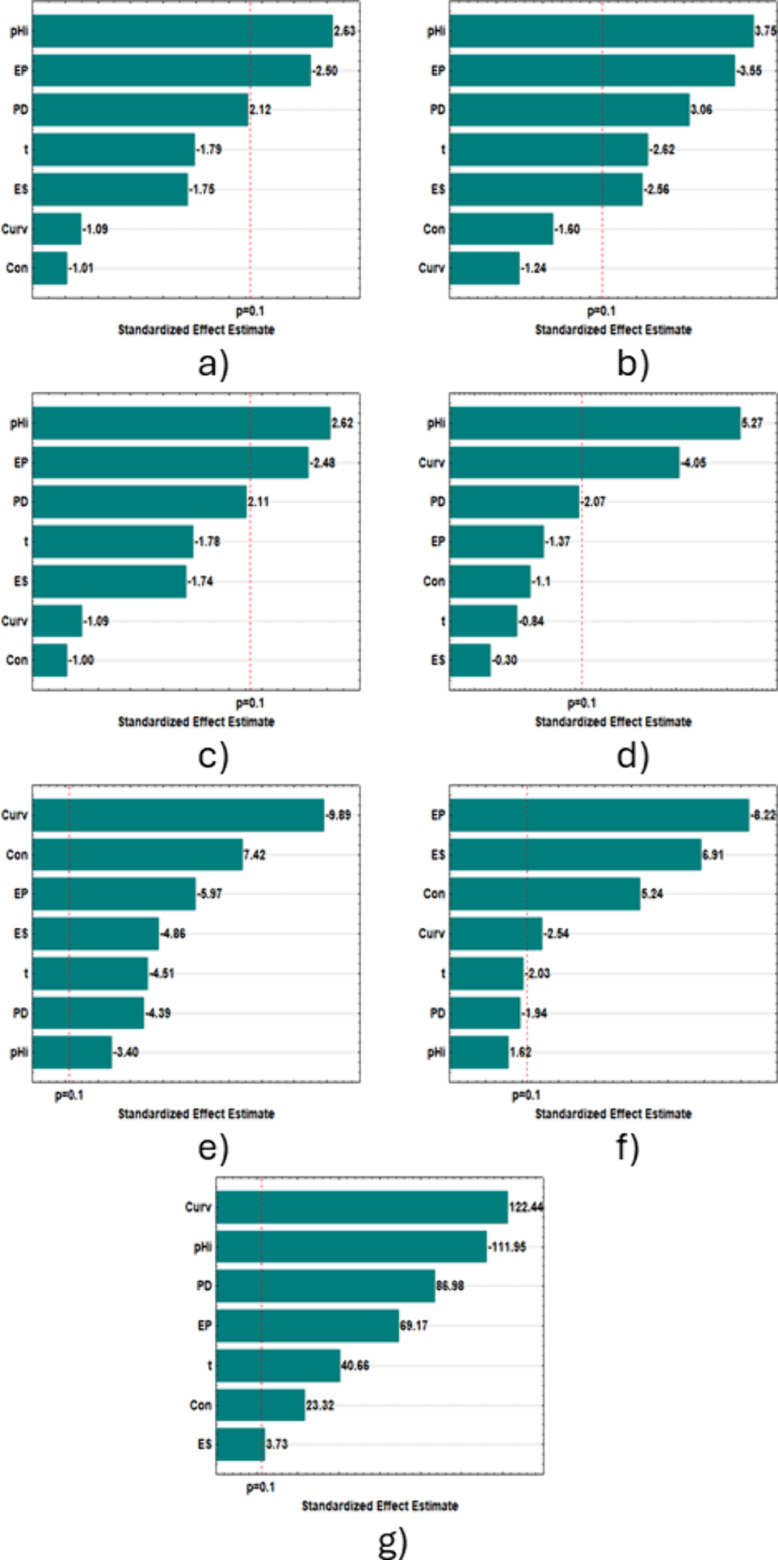
Pareto diagrams for FFD: a) conductivity (Λ); b) salinity (Sal); c) total dissolved solids (TDS); d) pH_f_; e) color (Col); f) turbidity (Tur), and g) removal rate (Rr).

The dye concentration, for instance, was not significant for conductivity, salinity, total dissolved solids, and final pH, but it was significant only for color, turbidity, and removal rate. The curvature effect was significant for final pH, color, turbidity, and removal rate, suggesting a potential quadratic model. The treatment time was significant for salinity, color, and dye removal rate. This indicates that expanding the working range of this factor may result in a significant mathematical model. Color and removal rate showed all effects significant at 90% confidence, highlighting the importance of the selected parameters as independent variables for the response variables that qualify the treatment performed.

Conductivity, total dissolved solids, and salinity are interdependent parameters because they all rely on the quantity of dissolved particles in the medium. In the EC treatment of textile effluents, dissolved solids may originate from the release of ions by the electrodes or from the addition of electrolytes during the dyeing process. Regarding the final conductivity of the effluent, most experiments exhibited a final conductivity (Table 3) that was either similar to or lower than the initial conductivity (Table 1). The release of Al^3+^ ions depends on the applied electrical tension, the sets of electrodes present, and the pH of the medium.^7,47–49^ These parameters contribute to changes in the final values of conductivity, salinity, and dissolved solids. Conductivity facilitates ion movement and helps reduce energy consumption. However, very high concentrations can increase the temperature of the treated solution, alter the flow of bubbles, increase the solubility of certain species, and raise electrode consumption.^47,51,52^

Reactions occurring at the electrodes continuously modify the pH of the medium. Opposite variations were observed for pH values: when the initial pH is low, it tends to increase significantly during electrolysis, and when the treatment starts with a high pH (pH 12), it tends to decrease reasonably.^49–53^ Simply put, pH increases due to the evolution of hydrogen gas at the cathode, generating hydroxide ions in the medium, which interact with aluminum ions to form insoluble hydroxides, leading to floc formation. In an essential medium, the system operates dynamically between consuming and producing hydroxides. While the cathode produces OH^–^ (eq 2), these ions react with metal cations, forming hydroxides that aid in contaminant removal.^40,49,53^

The most significant pH variation occurred during the experiments starting at pH 5. It is important to note that treatment was most effective in removal at pH 5 and/or 8.5, which aligns with literature data.^54^ However, very low pH can solubilize aluminum species, hindering floc formation and compromising removal. Additionally, it can interfere with ion migration and affect current values, leading to variations in the final process results. In most experiments, the final pH ranged from 5 to 9, meeting the requirements set by the Brazilian National Environmental Council.^55^

Color, turbidity, and pH are key parameters for classifying water bodies in Brazil, as outlined in CONAMA Resolution No. 430/2011.^55^ The presence of dissolved and suspended substances within water bodies can significantly impact their visual properties. Organic compounds, ions, industrial effluents, and other soluble or insoluble materials primarily contribute to these alterations.^56–58^ The treatment process demonstrated exceptional efficacy in color removal, with 100% color removal in some cases, 98% dye removal, and a remarkable 95% turbidity reduction (to 0.1 NTU), as evidenced by Table 3.

A central composite rotational design (CCRD) was employed to investigate the interactive effects of variables and identify optimal operating conditions. Given their statistical significance on at least one response variable, all independent factors were maintained in the model.

### Central Composite Rotational Design (CCRD)

The CCRD results for removing remazol red are presented in Table 4S. The system confirmed its excellent performance, showing satisfactory removals above 98% for trials 27, 32, and 40 and achieving 100% removal for experiment 25. ANOVA was applied to the response data to develop predictive models (Table S5). A confidence level of 90% was adopted for statistical significance, with p-values below 0.1 indicating significant effects. While p-values are essential, it is crucial to consider other factors, such as lack of fit, particularly regarding central points.^37,39,40^

**Table 4:** Experimental Outcomes at Optimized Conditions: Performance Metrics for Effluent Treatment

run	^a^ **Salg L** ^–1^	^b^ΛmS cm^–1^	^c^TDS ppm	^d^pH_f‑_	^e^Col (%)	^f^Tur NTU	^g^Rr%
**predicted**	**0.98**	**1.97**	**1.26**	**9.02**	**95.2**	**0.38**	**95.6**
**1**	0.86	1.76	1.13	8.83	95.23	0.52	96.28
**2**	1.05	2.1	1.34	9.25	96.82	0.55	93.45
**3**	1.01	2.03	1.30	8.81	93.45	0.62	93.78
^h^ **M**	**0.97**	**1.96**	**1.26**	**8.96**	**95.17**	**0.56**	**94.50**
^i^ **S**	0.096	0.179	0.115	0.248	1.686	0.051	1.547
^j^ **C**	0.97 ± 0.24	1.96 ± 0.45	1.26 ± 0.29	8.96 ± 0.62	95.17 ± 4.19	0.56 ± 0.13	94.5 ± 3.84
**relationship between the values obtained under experimental conditions and the legally established values for disposal**							
**variable**		**experimentally obtained value**		**maximum amount allowed for discard**		**references**	
^a^ **Sal/g L** ^**–1**^		0.987 (0.0987%)		<0.5% (salty water)		^55^	
^b^ **Λ/mS cm** ^**–1**^		1.96		5		^1,60^	
^c^ **TDS/ppm**		1.26		500		^43^	
^d^ **pH**_**f**_		9		9		^55,59^	
^f^ **Tur/NTU**		0.56		40 (fresh water quality standard)			
^e^ **Col/Pt L** ^**–1**^				75 mg Pt L^–1^		^28,55^	
**final** ^k^ **con/mg L** ^**–1**^		4.51		color absence*		^55^	

a Salinity.

b Conductivity.

c Total dissolved solids.

d Final pH.

e Color.

f Turbidity.

g Removal rate.

h Means.

i Standard deviation.

j Confidence interval.

k Concentration.

Salinity, conductivity, and total solids demonstrated adequate model fit, as indicated by a nonsignificant lack of fit (*p* > 0.1) and acceptable R^2^ values (Table S6). The responses for these variables followed a parametric distribution and aligned well with predicted values (Figures S2 and S3).

Relative error analysis (Figure 3) revealed excellent agreement between experimental and predicted values for final pH, with a relative error below 20% for all experiments and an R^2^ of 83.1%. In contrast, color, turbidity, and removal efficiency exhibited significant relative errors and poor model fit, hindering the development of reliable predictive models (Figure S3).

**Figure 3:**
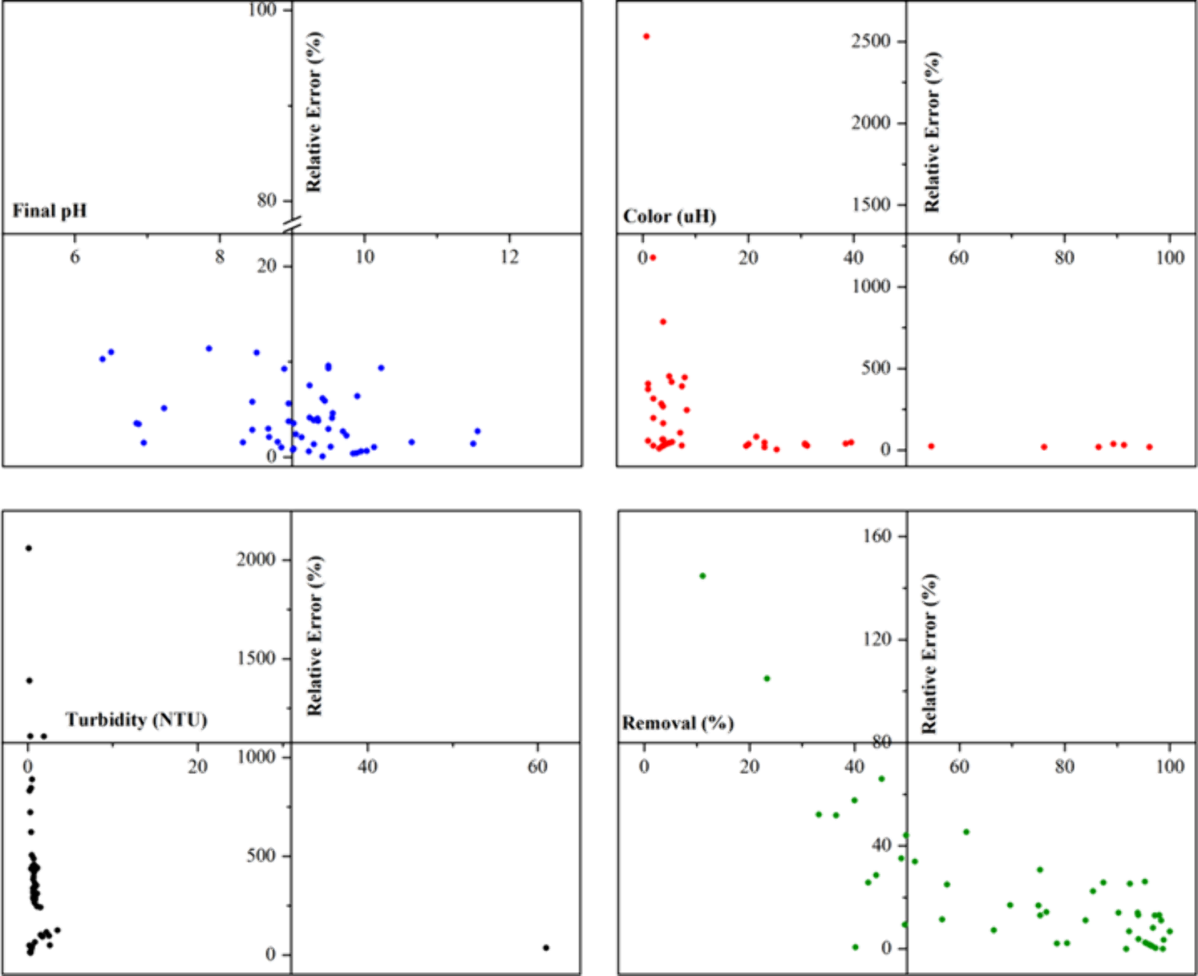
Relative error plots versus experimental values for the variables final pH, color, turbidity, and removal rate.

Despite these challenges, regression analysis was conducted, resulting in the adjusted mathematical models presented in eqs 12–15:

$$Salinity=0.892+0.112con+0.113{con}^{2}-0.082T{pH}_{i}$$ 12

$$Conductivity=1.809+0.204con+0.206{con}^{2}-0.152T{pH}_{i}$$ 13

$$Total\;solids=1.159+0,130con+0,132con^{2}-0,097TpH_{i}$$ 14

$$pH_{f}=8.940+0.327\;EP+0.102\;ES+0.231\;T+0.721pH_{i}+0.111\;con-0.473\;PDpH_{i}-0.135\;EPpH_{i}+0.202\;EP\;con-0.133\;ES\;T-0.228\;ESpH_{i}-0.284\;TpH_{i}+0.096\;T\;con+0.294pH_{i}con$$ 15

The most relevant fitted response surfaces obtained through CCRD are presented in Figure 4. These surfaces reveal key insights into the system’s behavior under different conditions. It can be observed that higher initial pH values significantly contribute to increased salinity and conductivity, as evaluated by the potential difference and electrode pairs (Figure 4a,b). Moreover, increased dye concentration increases total dissolved solids across electrode spacing and time duration (Figure 4e,f). A broad red region in the response surface indicates that high final pH values are likely if the effluent treatment begins with high initial pH levels (Figure 4h,i). As shown in Figure 2, other variables also play a crucial role in the effluent treatment process, and their interactions are essential for optimizing working conditions.

**Figure 4:**
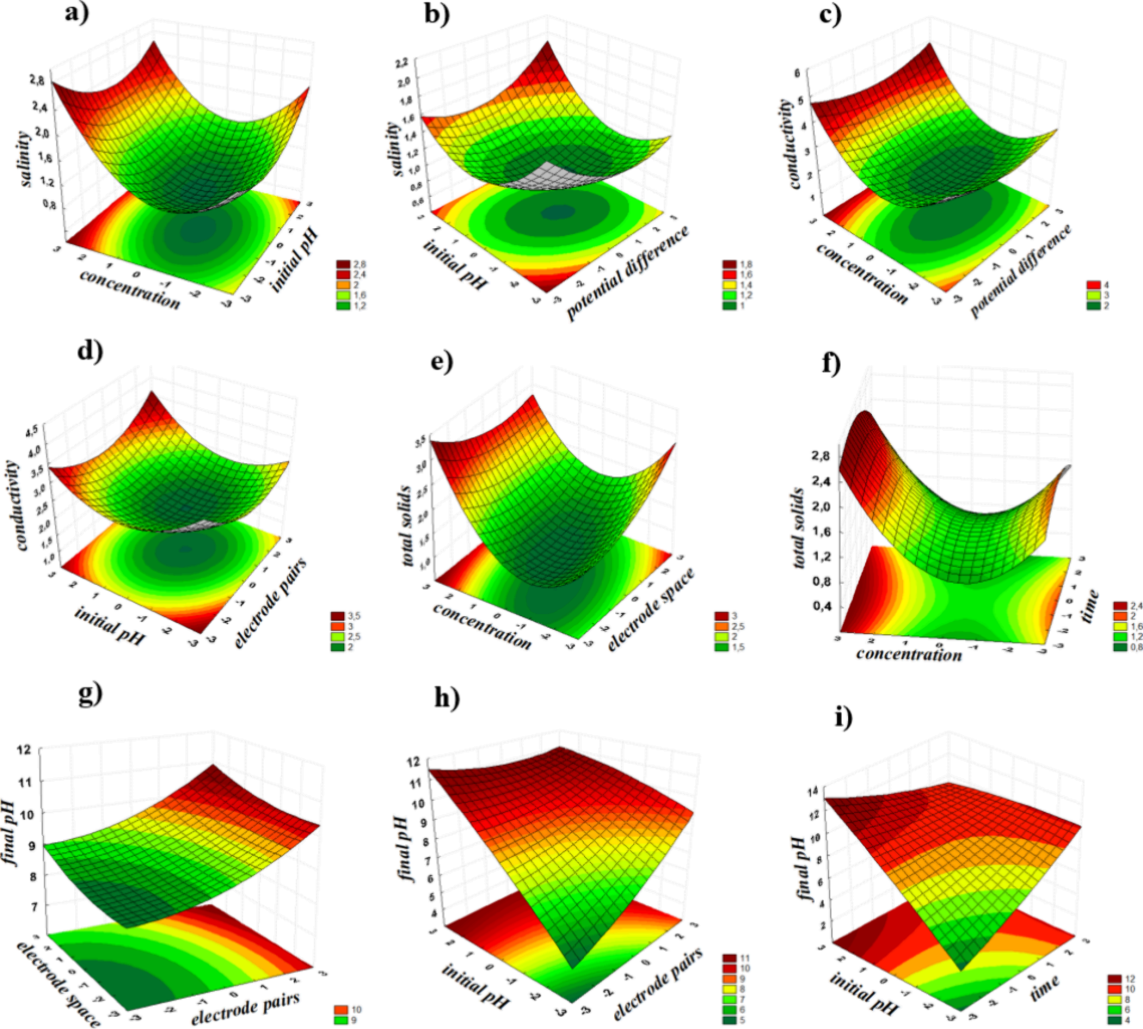
Response surface plots from the CCRD showing: (a) salinity vs initial dye concentration and initial pH; (b) salinity vs initial pH and applied potential; (c) conductivity vs dye concentration and applied potential; (d) conductivity vs initial pH and electrode pairs; (e) total solids vs dye concentration and electrode spacing; (f) total solids vs dye concentration and electrolysis time; (g) final pH vs electrode spacing and electrode pairs; (h) final pH vs initial pH and electrode pairs; and (i) final pH vs initial pH and electrolysis time.

### Optimized Experimental Conditions

The desirability function analysis identified the optimal electrocoagulation (EC) conditions as 12.7 V potential difference, 2.02 electrode pairs, 2.02 cm electrode spacing, 76.2 min electrolysis time, initial pH 8.4, and dye concentration of 81.9 mg L^–1^, as detailed in Figure 5. Under these conditions, the model achieved maximum desirability for all response variables (Table 4), and validation experiments confirmed the strong agreement between predicted and experimental values. These results demonstrate the reliability of the proposed mathematical model and its ability to guide process optimization effectively.

**Figure 5:**
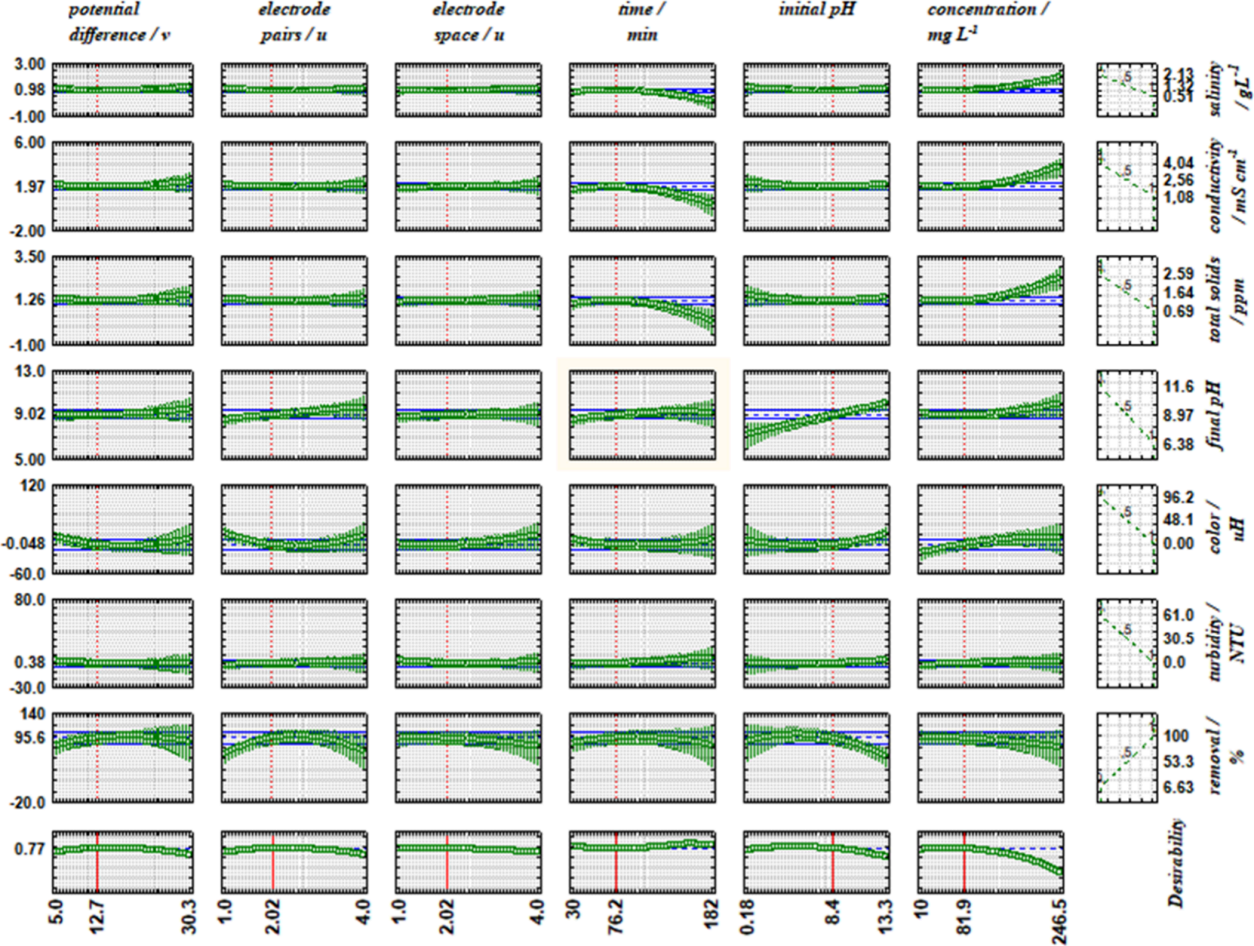
Predicted and desirability profiles for key effluent treatment metrics: salinity, conductivity, total dissolved solids, final pH, color, turbidity, and removal.

At the optimized point, the EC system achieved a dye removal efficiency of 94.50% and a color removal of 95.17%, with a final pH of 8.96 and residual turbidity of only 0.56 NTU. These performance levels comply with Brazilian effluent discharge standards and confirm the suitability of EC for textile wastewater treatment.^44,59^ The high removal rates indicate near-complete decolorization of Remazol Red, primarily through coagulation, adsorption, and electroflotation mechanisms promoted by the aluminum electrodes. during electrolysis, which helps stabilize the process and reduces the need for post-treatment pH adjustment.^19,48,61,62^

### Electrode Consumption and Efficiency of the EC Process

The mass of the oxidized electrodes was determined experimentally by measuring the difference in electrode mass before and after each trial. The results of these analyses for the trials predicted to have the highest efficiency (optimized CCRD conditions: V = 12 V; EP = 2; ES = 2 cm; t = 76 min; initial pH = 8.4, and dye concentration = 82 mgL^–1^) are presented in Table S7.

Faraday’s laws can theoretically calculate the mass of the material (aluminum) consumed electrolytically during the process. According to eq 16,^63–65^ the mass consumed from the electrode (m, in grams) can be determined using the parameters: i (current, A), t (time, s), and n (number of electrons involved), in addition to the molar mass of the anode (Al, 27.0 gmol^–1^) and Faraday’s constant (F, 96,485.3 C mol^–1^). The current values and the corresponding theoretical consumption of Al are also presented in Table S8.

$$m=\frac{i.t.M}{F.n}$$ 16

Due to the nature of the electrode and its oxidation potential, the experimental values for mass loss were consistently higher than those predicted by Faraday’s law. This discrepancy is attributed to the additional wear or consumption of the aluminum electrode, resulting from the presence of H^+^_(aq)_ or OH^–^_(aq)_ ions in the medium, which form species dependent on the pH value.^62–65^

The energy consumption of the process (Ce, Wh m^–3^) can be calculated using eq 17,^66^ where U is the voltage applied (V); i is the current (A); t is the electrolysis time (h); and V is the volume of treated effluent (m^3^).

$$C_{e}=\frac{U.i.t}{V}$$ 17

The results obtained are presented in Table 8S.

To better contextualize the findings of the present work, a comparative analysis was performed against **other** recent EC studies involving both synthetic dye solutions and real textile effluents (Table 5). Our research focused on the electrocoagulation (EC) removal of Remazol Red under optimal conditions determined through experimental design. The high efficiency achieved in this study demonstrates the strong potential of EC for textile wastewater treatment. Under the optimal conditions, the system achieved 94.50% dye removal and 95.17% color removal, which is highly competitive. However, when comparing with synthetic azo dyes, our dye removal rate is slightly lower than the nearly complete removal reported for Reactive Red 43 (99.8%)­42, though higher than those achieved for Methylene Blue (82%)^12^ and Methyl Orange (81%).^12^ These differences may be attributed to the specific molecular structure of Remazol Red or to the moderate operational conditions employed in our experimental design.

**Table 5:** Performance Comparison of Aluminum Electrode Electrocoagulation (EC-Al) for the Treatment of Various Dyes and Textile Effluents.

dye/wastewater characteristics	optimal conditions	key removal results	ref
methylene blue/10 to 60 mg L^–1^, pH 4 to 10, cond. 3 mS/cm	60 mg L^–1^, pH 8, 15 V, 60 min, 2 cm electrode spacing	82% dye removal	^12^
methyl orange/10 to 60 mg L^–1^, pH 4 to 10, cond. 3 mS cm^–1^	60 mg L^–1^, pH 8, 15 V, 60 min, 2 cm electrode spacing	81% dye removal	^12^
real textile wastewater/pH 13, cond. 5.56 mS cm^–1^, TDS 4,95 mg L^–1^, salinity 4.05 g L^–1^, COD 720 g/L, TSS 420 mg L^–1^, turb. 30 NTU	pH 7–8, 15 V, 60 min, 20-min settling time	86% dye removal, TSS 75%, COD 79%, TDS 91%	^12^
real textile dyeing wastewater/pH 11.5, cond. 22.5 mS cm^–1^, COD 1,450 mg L^–1^, TSS 280 mg L^–1^, turb. 230 NTU, color 1765 Pt-Co, BOD_5_ 520 mg L^–1^, chlorides 4,200 mg L^–1^	pH 9, 36.26 min, 4 V, 57.1 A m^–2^, 1 cm interelectrode distance	COD 63.05%, 99.07% dye removal, turbidity removal 96.31%, operating cost 0.4705 USD m^–3^ energy consumption = 0.316 kWh m^–3^	^15^
methylene blue/5.12 mg L^–1^, pH 2 to 9, turb. <2.5 NTU, color 207–302 uH, KCl 745.5 mg L^–1^	pH 4, 32 V, 203 min, 4 pairs of electrodes, 2.5 cm interelectrode distance	76% volor removal, 95.1%: dye removal, energy consumption: 32.5 kWh L^–1^	^22^
real textile effluent (postbiological discharge from a dyeing industry)/pH 9.0, cond. 1.5 mS cm^–1^, color 720 mg PtCo L^–1^, turb. 120 NTU, COD 105 mg L^–1^, TSS 18 mg L^–1^	pH 3, 200 A m^–2^, 3 cm interelectrode distance, 15 min, voltage: up to 30 V	92% color removal,82% aromatic compounds, 82% turbidity, 64% COD	^24^
real textile effluent/pH 7.7, cond. 5.5 mS cm^–1^, TDS 3.575 mg L^–1^, COD 512 mg L^–1^, turb. 206 NTU, color 1096 Pt-Co	pH 6.0, 15 V, 7.9 mA cm^–2^, 40 min	95.4% color remotion, 92.5% COD removal, 97.6%, turbidity removal, energy consumption: 1.12 kWh m^–3^, operating cost: 0.013 USD m^–3^	^28^
reactive red 43/100 mg L^–1^, pH 3.31 to 9.68, cond. 2.8 mS cm^–1^, NaCl 1580 to 6540 mg L^–1^	pH 6.0, 15 V, 6.3 A m^–2^, 30 min	99.8% color remotion, 84% COD removal, energy consumption: 3.9 kWh m^–3^, operating cost: 0.0056 USD m^–3^	^42^
remazol red/dye concentration 10 to 150 mg L^–1^, pH 5.0 to 12.0, cond. < 2.6 mS cm^–1^, turb. < 2.0 NTU, NaCl 1,0 g L^–1^	pH 8.4, dye concentration 82 mg L^–1^, 12.7 V, 76.2 min	95.17% color removal, 94.50% dye removal: energy consumption: ≈ 4.70 kWh m^–3^	this work

When compared with studies on the more demanding treatment of real textile effluents, our color removal efficiency (95.17%) remains comparable to or higher than the values reported by Núñez et al.^28^ (95.4%) and Gasmi et al.^15^ (99.07%), and clearly exceeds the 92% color removal obtained by Menon et al.^24^ for postbiologically treated wastewater. These results further validate the effectiveness of the EC process, particularly with aluminum electrodes, in handling the complexity of reactive dye systems, even under conditions not fully optimized for COD removal.

The technical feasibility of EC processes is strongly influenced by their energy footprint. The energy consumption calculated in this study (≈4.70 kW h m^–3^) falls within the typical range reported for textile effluent electrocoagulation. Although this value is higher than that of the highly efficient system described by Núñez et al.28 (1.12 kW h m^–3^), the difference is likely due to the longer electrolysis time employed here (76.2 min vs 40 min) and potential variations in current density, which directly affects power demand. Furthermore, the energy consumption (C_e_ values obtained are consistent with the data reported in the literature. Oliveira,^67^ for example, reported a C_e_ value of 4.56 kW h m^–3^ for an aluminum electrode and a color removal efficiency of 81.9%. The same author demonstrated that these values can be improved by modifying the nature and structure of the electrode (e.g., nitriding).

In Brazil, the cost of electricity for the industrial sector remains relatively high compared to other countries such as France, Mexico, Turkey, and Canada. Recently, Brazil ranked unfavorably, given its internal potential, at 113th place worldwide in industrial electricity tariffs, measured in USD/MW h.^68^ The average rate recorded for August 2021 was 684.77 reais per MW h, which is approximately 0.685 reais per kWh for this sector.^68^ The process cost per cubic meter of effluent is presented in Table 8S. Considering the potential for alternative energy sources (e.g., solar or wind power), these costs could become more competitive, highlighting the potential of this technique.

Thus, the combination of competitive removal efficiency and moderate energy consumption (≈4.70 kWh m^–3^) demonstrates the techno-economic feasibility of the optimized EC system for the treatment of Remazol Red and analogous textile wastewaters, laying a strong foundation for future scale-up studies.

### Analysis of Aluminum Electrode Composition, Effluents, and Generated Residues

Figure S4 in the SM shows a visual comparison of an effluent sample before and after treatment using the optimized parameters. It highlights the significant improvement in water quality and the produced sludge, which exhibits a color similar to the original dye, suggesting removal by adsorption.

In addition to measurements of salinity, conductivity, total dissolved solids (TDS), pH, color, turbidity, and removal efficiency (Table 4), the treated effluent was analyzed for its Al^3+^ ion content using AAS and subjected to HPLC analysis (Figures S5 and S6 and Table S9).

AAS analysis revealed an Al^3^^+^_(aq)_ concentration of 0.92 mg L^–1^ in the treated effluent. Most studies do not evaluate this parameter in the treated effluent, complicating accurate comparisons. However, as aluminum is amphoteric and has a highly negative reduction potential, it is important to note that the aluminum concentration is likely dependent on the pH used during treatment. For an initial pH of 8.4, the observed concentration aligns with literature data, although some studies^69,70^ have reported lower dissolved aluminum concentrations. For example, Sangal et al.^70^ worked with an initial pH of 6.5 and, using an ICP spectrophotometer found a residual concentration of about 0.001 mg L^–1^. However, at higher initial pH values (7.16–7.20), Aitbara et al.*,* ^29^ working with a variable current density (8.8–23.8 mA cm^–2^), reported a residual aluminum concentration of 93.03–465.69 mg L^–1^ for pure Al electrodes and 102.26–502.48 mg L^–1^ for Al alloy electrodes. Cell arrangements employing cathodes made of pH-resistant materials, such as Cu, reduce the residual Al^3^^+^_(aq)_ concentration while minimizing electrode wear.^71^

From a regulatory standpoint, the soluble aluminum content of 0.92 mg L^–1^ exceeds the limit for direct discharge into high-quality freshwater bodies but remains suitable for controlled industrial reuse. In Brazil, effluent management is governed by CONAMA Resolution No. 357/2005,^72^ which classifies surface waters and sets quality standards.

For Class 1 or 2 freshwater bodies, the maximum allowable dissolved aluminum concentration is 0.1 mg L^–1^, as established by CONAMA 357 itself. It is important to clarify that the Ministry of Health’s Ordinance GM/MS No. 888/2021,^73^ which defines potability standards, sets a limit of 0.2 mg L^–1^ for aluminum in drinking water, but this standard is distinct from those for environmental discharge. CONAMA Resolution No. 430/2011 ^55^ stipulates that effluents must not alter the classification of the receiving body, meaning they must comply with the standards set by CONAMA 357/2005. Therefore, for discharge into Class 1 or 2 freshwater, the Al^3^^+^ concentration must not exceed 0.1 mg L^–1^.

Consequently, the Al^3^^+^ concentration of 0.92 mg L^–1^ observed in this study is not suitable for direct discharge into these protected systems without additional polishing. However, it is fully compatible with nonpotable reuse applications, such as industrial operations (e.g., textile processes or cooling systems), fertigation, or closed-loop recycling. These strategies mitigate environmental impact while leveraging the high decolorization efficiency and moderate energy consumption of the optimized EC process. Moreover, industrial water reuse is a crucial step toward sustainable textile manufacturing. As highlighted in recent studies, recycling treated effluent can significantly reduce freshwater consumption (e.g., up to 300 m^3^ day^–1^ in typical mills) and alleviate pressure on water resources, particularly in water-scarce regions.^15^ This approach enhances both the environmental and economic sustainability of the EC process.

HPLC analysis (Figure S6) confirmed the dye’s removal after treatment. The chromatogram of the untreated synthetic effluent displayed a single peak at a retention time of 3.97 min, corresponding to the dye, which was absent in the chromatogram of the treated effluent. This result demonstrates the efficiency of the removal process. Additionally, the untreated effluent’s chromatogram revealed the commercial dye’s relative purity. The findings confirm that no primary dye degradation products were detected above the method’s limit of detection (LOD), indicating that physical removal via coagulation was the predominant mechanism. While the formation of trace transformation byproducts cannot be entirely ruled out, the efficient elimination of the parent Remazol Red molecule remains the most significant outcome for assessing potential elution risks and reuse feasibility.

The aluminum content was also determined for electrode samples (fabricated using aluminum plates acquired from the local market) and the sludge obtained after treatment. Figure S5 illustrates one of the analytical calibration curves obtained for these analyses, and the results are presented in Table S9.

The analysis confirmed the quality of the aluminum electrode material (99.80%). This implies that for calculating electrode mass loss due to consumption, as performed earlier, the wear of the electrodes can be considered solely as aluminum oxidation without needing corrections for electrode composition.

The anode was also characterized by SEM and EDX, both before and after use. Figure 6 shows the micrographs obtained for the anode, and Table 6 and Figure 7 present the compositional results and corresponding spectra.

**Figure 6:**
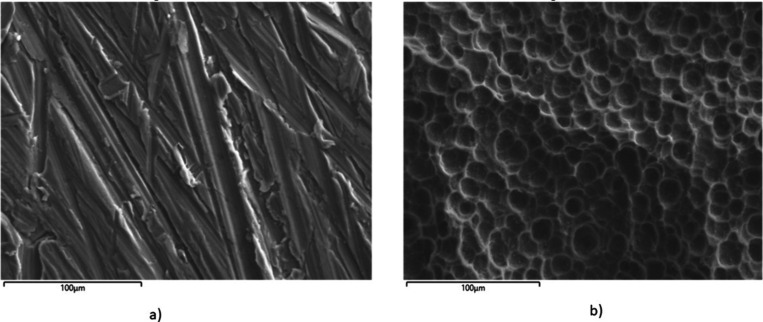
Micrographs of the Al electrode (anode) a) before use; b) After use.

**Table 6:** Composition of Aluminum Electrode (Anode): a) before Use; b) after Use, (n = 3).

	anode chemical composition (% by weight)	
element	a)	b
O	2.89 ± 0.40	8.16 ± 1.20
Al	96.64 ± 0.80	90.68 ± 1.30
Fe	0.47 ± 0.05	1.16 ± 0.12

**Figure 7:**
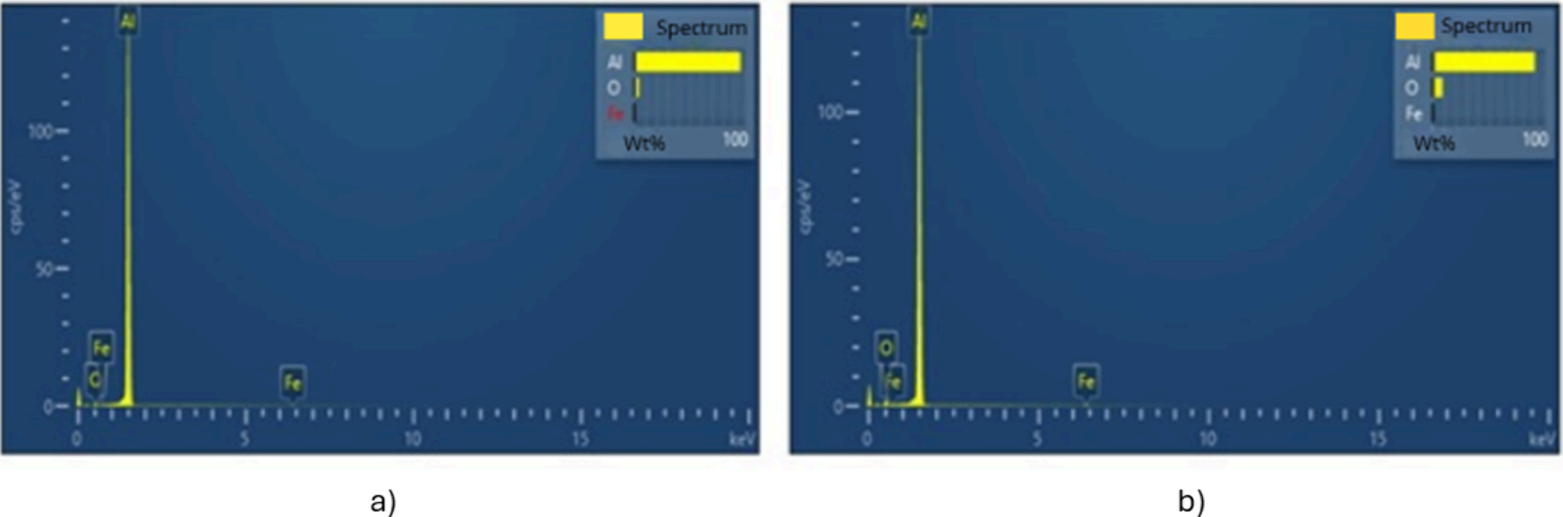
EDX spectra of the aluminum electrode (anode): a) before use and b) after use.

The micrographs reveal notable alterations on the anode’s surface after use. Initially, grooves due to the aluminum rolling process are visible on the plates, followed by the corroded surface of the anode, displaying vortices or pits that indicate a specific wear pattern and metal release.

As expected, differences were also observed regarding composition between the results obtained by AAS after sample digestion and the EDX data for the electrode before use. The EDX measurements are restricted to the electrode’s surface, where the percentage of oxygen is significantly higher due to the spontaneous formation of Al_2_O_3_ across the metal surface, inhibiting further and more profound oxidation of the metal.

Table 6 shows that a small amount of Fe is present in the electrode. As previously mentioned, Fe is also commonly used as an electrode for electrocoagulation, so the presence of this metal in the anode is not problematic. The surface composition of the electrode after use reveals an increased content of oxidized species, which is expected due to the medium’s characteristics, favoring hydroxide formation.

The sludge’s aluminum content was 23% w/w (Table S9). One of the previously highlighted advantages of electrocoagulation (EC) technology applied to wastewater treatment is the small amount of sludge generated compared to other methodologies.^8,56^ Figure S4 shows the sludge obtained after drying and grinding, where the dye’s presence is evidenced by its coloration. Figure S7b presents the X-ray diffractogram of the sludge produced during the process. The diffractogram exhibits broad peaks, characteristic of materials with low crystallinity, with maxima located at 2θ = 13.9°, 28.4°, 38.8°, 49.3°, 65.1°, and 71.4°. These patterns are consistent with aluminum oxyhydroxide AlO­(OH) (PDF 49–133), as observed in Figure S7a. The presence of broad peaks indicates a partially crystalline or amorphous structure, possibly influenced by water retention (see FTIR and TG) and the complex composition of the sludge.^74^

The dye’s diffractogram is also illustrated in Figure S7c. According to Souza,^74^ the crystallinity observed in the dye’s diffractogram is due to the presence of impurities, notably Na_2_SO_4_ (PDF 37–1465), NaCl (PDF 75–0306), and sulfur (PDF 42–1278). The presence of Na_2_SO_4_, NaCl, and sulfur explains the high-intensity peaks observed at: 2θ = 19.3°, 34.4°, 49°, and 56.8° (Na_2_SO_4_); 26.1° and 38.9° (sulfur); and 32.1° and 45.8° (NaCl). However, the dye’s most intense peaks are not observed in the sludge, as the salts responsible for the dye’s crystallinity likely remain soluble in the effluent.

Chromatographic analysis of the effluent before electrocoagulation revealed only a single peak corresponding to the dye (retention time of 3.97 min), as illustrated in Figure S6. These results indicate the stability and quality of the dye used as a model.

The sludge and the dye were characterized using FTIR and TG techniques for comparative analysis. The results are presented in Figures S8 and S9, respectively. For the Remazol Red dye (Figure S8a), the main vibrational modes were assigned as follows: ν­(OH) in the range of 3600–3100 cm^–1^, due to the presence of water; 1547 cm^–1^ – ν­(C-N)_sym_; 1657–1478 cm^–1^ ν­(C = C)_ring_; 1040 cm^–1^ – indicative of the S = O bond in sulfonate groups; and 1389 and 1289 cm^–1^ – ν­(C-N)_sym_ and ν­(C-N)_asym_ in the aromatic ring, respectively.^74^

For the sludge (Figure S8b), the observed vibrational modes were more consistent with the presence of aluminum oxyhydroxide than with the adsorbed dye. The identified peaks include: 3304 cm^–1^ (ν­(OH)), attributed to both hydroxyl groups and adsorbed water; 1637 cm^–1^ (δ­(OH_2_)) from adsorbed water; 1058 cm^–1^ (δ­(OH) or ν­(Al-O); and 469 cm^–1^ (ν­(Al-O)).^75–78^

The results of the TGA and DTG analyses are summarized in Table S10. As reported by Souza,^74^ the TGA curve of the dye (Figure S9a) exhibited three main events under synthetic air: (i) dehydration between 50 and 205 °C (T_onset_ ≈ 50 °C; T_max_ = 107 °C; Δm = 6.3%; DTG_max_ = – 0.11% °C^1–^), attributed to the loss of adsorbed and bound water; (ii) thermal degradation between 206 and 402 °C (T_onset_ ≈ 230 °C; T_max_ = 326 °C; Δm = 10.0%; DTG_max_ = – 0.69% °C^1–^), corresponding to the decomposition of the dye’s organic framework; and (iii) decomposition and carbonization between 403 and 800 °C (T_onset_ ≈ 402 °C; T_max_ = 530 °C; Δm = 36.5%; DTG_max_ = – 0.34% °C^1–^), yielding a carbonaceous residue of 51.2%. The progressively lower T_onset_

The sludge (Figure S9b) also displayed three distinct thermal events. The first event (50–230 °C; T_onset_ ≈ 50 °C; T_max_ = 150.6 °C; Δm = 16.5%; DTG_max_ = – 0.24% °C^1–^) revealed a higher mass loss than the dye, due to desorption of physically adsorbed water and volatilization of trapped organic residues, including residual dye. This is consistent with Kloprogge et al.,^75^ who reported water loss in aluminum oxyhydroxides (gibbsite, boehmite, diaspore) within this temperature range, though with smaller weight changes (∼2%). The second event (231–520 °C; T_onset_ ≈ 310 °C; T_max_ = 393 °C; Δm = 18.7%; DTG_max_ = – 0.09% °C^1–^) corresponds mainly to the degradation of flocculated organic matter and partial dehydroxylation of aluminum oxyhydroxide, as described by the reaction 2AlO­(OH) → Al_2_O_3_ + H_2_O (theoretical mass loss ≈15%).

Finally, the third region (521–800 °C; T_onset_ ≈ 520 °C; Δm = 8.9%; DTG_max_ = – 0.05% °C^1–^) exhibited reduced thermal activity, with the absence of a pronounced DTG peak, indicating that most reactive organic species had already decomposed. The lack of a distinct decomposition peak near 530 °C, observed in the pure dye, suggests that the dye molecules in the sludge underwent transformation during the electrocoagulation process. Despite the residual coloration of the sludge, the combined TG–DTG results indicate that its composition is dominated by hydrated aluminum oxyhydroxides and carbonaceous species rather than undegraded dye. These results, supported by both TG and DTG analyses, confirm that the sludge composition is dominated by hydrated aluminum oxyhydroxides and carbonaceous residues, with the organic dye likely incorporated or complexed within the inorganic framework formed during electrocoagulation.

In addition to the limited volume generated, a quantitative sludge mass balance established that the electrocoagulation process produced 0.175 kg of dry solids per m^3^ of treated wastewater, placing it within the lower range reported in literature (0.16–1.44 kg m^–3^).^15^ Physicochemical and thermal analyses confirmed that this sludge consists predominantly of amorphous aluminum (oxy)­hydroxides with minor carbonaceous residues and negligible amounts of undegraded dye, indicating a chemically stable, nonhazardous material suitable for valorization.

The management perspective for EC sludge has consequently shifted from waste disposal to resource recovery, aligning with circular economy principles. Given its valuable aluminum content and reactive surface sites, this EC sludge can be repurposed as raw material for adsorbent synthesis, construction materials, or secondary coagulants after calcination.^79^ Such valorization pathways, including application in ceramics or as coagulant aids, not only reduce disposal demands but also mitigate operational costs while enhancing process sustainability.

As emphasized by Gasmi et al.,^15^ both treated effluent and sludge from EC systems can be reintegrated into industrial processes through water recycling or transformation into useful byproducts. These integrated strategies demonstrate that electrocoagulation operates not merely as a treatment technology but as a comprehensive resource recovery platform, reducing environmental impact and promoting sustainable textile manufacturing.

## Conclusion

Electrocoagulation effectively removed Remazol Red dye from synthetic effluents using aluminum electrodes under low-voltage conditions, achieving up to 100% dye removal depending on initial concentration. Optimal parameters for higher concentrations were identified by the desirability function: 82 mg L^–1^ dye, 12.7 V, 2 electrode pairs, 2 cm spacing, 76.2 min, and pH 8.4. Predicted outcomes included salinity (0.98 g L^–1^), conductivity (1.97 mS cm^–1^), TDS (1.26 mg L^–1^), final pH (9.02), turbidity (0.38 NTU), color removal (95.2%) and dye removal (95.6%). Experimental results (n = 3) closely matched these predictions: salinity (0.97 g L^–1^), conductivity (1.96 mS cm^–1^), TDS (1.26 mg L^–1^), final pH (8.96), turbidity (0.56 NTU), color removal (95.17%), and dye removal (94.50%).

Confirmation experiments demonstrated no statistically significant differences (*p* > 0.05 at 95% confidence), thereby confirming that the model built using a 90% confidence level for variable selection provided robust and reliable predictions within the studied range. Electrode and energy consumption under optimal conditions aligned with literature data, energy usage averaging 4.70 kWh m^–3^ and cost estimated at 3.22 Brazilian reals (BRL) per m^3^. HPLC analyses of treated effluent showed no detectable dye, while AAS indicated residual Al^3^^+^ at 0.92 mg L^–1^, potentially critical depending on discharge regulations. The dried sludge contained 23.3% w/w aluminum (AAS). XRD, FTIR, and TGA confirmed AlO­(OH) (aluminum oxyhydroxide) as the main phase, convertible to alumina (Al_2_O_3_) above 293 °C. EC-DAF performance did not significantly differ from EC alone under the tested conditions and cell configuration. Overall, results confirm electrocoagulation as an effective and sustainable method for treating textile effluents, which are challenging due to their solubility, chemical stability, and environmental persistence.

## Supplementary Material



## Notes

Available online: Experimental design matrix (CCRD); ANOVA tables; optimization plots; validation of statistical models; residual and normal probability plots; additional electrochemical data; FTIR spectra; XRD diffractograms; TGA/DTG curves; HPLC chromatograms; and supplementary figures and tables (Figures S1–S10, Tables S1–S12) (PDF)

## References

[1] Gasmi A., Elboughdiri N., Ghernaout D., Hannachi A., Halim K. S. A., Khan M. I. (2022). Electrocoagulation Process for Removing Dyes and Chemical
Oxygen Demand from Wastewater: Operational Conditions and Economic
Assessment – a Review. Desalin. Water
Treat..

[2] do
Vale-Júnior E., da Silva D. R., Fajardo A. S., Martínez-Huitle C. A. (2018). Treatment
of an Azo Dye Effluent by Peroxi-Coagulation and its Comparison to
Traditional Electrochemical Advanced Processes. Chemosphere.

[3] Friede R. (2020). Aumento Populacional
e Degradação Ambiental: a Aonta que Não Quer
Fechar. Rev. Augustus.

[4] Gürses, A. ; Güneş, K. ; Şahin, E. Removal of Dyes and Pigments from Industrial Effluents. In Green Chemistry and Water Remediation: Research and Applications; Sharma, S. K. ; Mudhoo, A. , Eds.; Elsevier: Amsterdam, 2021; pp 135–187. DOI: 10.1016/B978-0-12-817742-6.00005-0.

[5] Casas G., Martinez-Varela A., Vila-Costa M., Jiménez B., Dachs J. (2021). Rain Amplification
of Persistent Organic Pollutants. Environ. Sci.
Technol..

[6] Titchou F. E., Zazou H., Afanga H., El Gaayda J., Ait Akbour R., Hamdani M. (2021). Removal of Persistent Organic Pollutants
(POPs) from Water and Wastewater by Adsorption and Electrocoagulation
Process. Groundwater Sustainable Dev..

[7] Titchou F. E., Afanga H., Zazou H., Ait Akbour R., Hamdani M. (2020). Batch Elimination of Cationic Dye
from Aqueous Solution
by Electrocoagulation Process. Groundwater Sustainable
Dev..

[8] AlJaberi F. Y. (2019). Operating
Cost Analysis of a Concentric Aluminum Tubes Electrodes Electrocoagulation
Reactor. Heliyon.

[9] Can O. T., Bayramoglu M., Kobya M. (2003). Decolorization of Reactive
Dye Solutions
by Electrocoagulation Using Aluminum Electrodes. Ind. Eng. Chem. Res..

[10] Schillemans T., Yan Y., Ribbenstedt A., Donat-Vargas C., Lindh C. H., Kiviranta H., Rantakokko H., Wolk A., Landberg R., Åkesson A., Brunius C. (2024). OMICs Signatures Linking Persistent Organic Pollutants
to Cardiovascular Disease in the Swedish Mammography Cohort. Environ. Sci. Technol..

[11] Koenig S., Huertas D., Fernández P. (2013). Legacy and
Emergent Persistent Organic
Pollutants (POPs) in NW Mediterranean Deep-Sea Organisms. Sci. Total Environ..

[12] Zafar A. M., Naeem A., Minhas M. A., Hasan J., Rafique S., Ikhlaq (2024). Removal of Reactive
Dyes from Textile Industrial Effluent Using Electrocoagulation in
Different Parametric Conditions of Aluminum Electrodes. Environ. Adv..

[13] Rekha H., Murthy U. (2018). Electrochemical Degradation of Remazol
Red RB 133 Using
Sacrificial Electrodes. Int. J. Sci. Technol..

[14] Pavithra M. P., Pushpa L. (2020). Treatment of Leachate
by Electrochemical Oxidation
Using Graphite and Titanium Electrodes. Samriddhi
– J. Phys. Sci. Eng. Technol..

[15] Gasmi A., Ibrahimi S., Elboughdiri N., Tekaya M. A., Ghernaout D., Hannachi A., Mesloub A., Ayadi B., Kolsi L. (2022). Comparative
Study of Chemical Coagulation and Electrocoagulation for the Treatment
of Real Textile Wastewater: Optimization and Operating Cost Estimation. ACS Omega.

[16] Ahlawat K., Jangra R., Prakash R. (2024). Environmentally
Friendly UV-C Excimer
Light Source with Advanced Oxidation Process for Rapid Mineralization
of Azo Dye in Wastewater. ACS Omega.

[17] Sterenzon E., Vadivel V. K., Gerchman Y., Luxbacher T., Narayanan R., Mamane H. (2022). Effective Removal of Acid Dye in
Synthetic and Silk Dyeing Effluent: Isotherm and Kinetic Studies. ACS Omega.

[18] Khan S., Noor T., Iqbal N., Yaqoob L. (2024). Photocatalytic
Dye
Degradation from Textile Wastewater: A Review. ACS Omega.

[19] Zaldivar-Díaz J. M., Martínez-Miranda V., Castillo-Suárez L. A., Linares-Hernández I., Ríos M. J. S., Alcántara-Valladolid A. E. (2023). Synergistic
Electrocoagulation-Precipitation
Process Using Magnesium Electrodes for Denim Wastewater Treatment:
Bifunctional Support Electrolyte Effect. J.
Water Process Eng..

[20] Bezerra, W. B. ; Nunes, G. P. S. M. ; Goes, M. C. D. C. ; Cruz, F. F. D. S. D. ; Nascimento, U. M. ; Santana, C. G. D. ; Cardoso, J. J. F. ; Santana, S. A. A. ; Melo, S. M. ; Dendasck, C. V. ; Messeder, J. C. Simplified Electrocoagulation for Efficient Biodiesel Washing Water Treatment. In Revista Científica Multidisciplinar Núcleo do Conhecimento; 2023, Vol. 2, pp. 136-165. DOI: 10.32749/nucleodoconhecimento.com.br/quimica-en/simplified-electrocoagulation.

[21] de
Carvalho Góes M. C., Garcez M. P. R., Siqueira A. R. F., Farias T. P., de Santana C. G., de Jesus Gomes da Costa
Neto J., Bezerra C. W. B. (2021). Pectin and SDS as Auxiliary Flocculants
for Complementary Treatment of Textile Wastewater by Electrocoagulation. Korean J. Chem. Eng..

[22] Góes M., Garcez M., Santana C., Costa Neto J., Bezerra C. (2021). Sequential Design of Experiments for Removal of Methylene
Blue Dye by Electrocoagulation Associated with Dissolved-Air System. J. Braz. Chem. Soc..

[23] Schwarzenbach R. P., Egli T., Hofstetter T. B., von Gunten U., Wehrli B. (2010). Global Water Pollution and Human
Health. Annu. Rev. Environ. Resour..

[24] Carina
Menon B., Rubens Lapolli F., Angeles Lobo-Recio M., Eliza Nagel-Hassemer M. (2020). Evaluation of the Electrocoagulation Process in the
Treatment of Textile Effluents. Rev. DAE.

[25] Tomassoni F., Giroletti C. L., Dalari B. L. S. K., Nagel-Hassemer M. E., Recio M. A. L, Lapolli F. R. (2019). Optimization
of Electrocoagulation
Applied to Textile Effluent. Revista DAE.

[26] Cotillas S., Llanos J., Cañizares P., Clematis D., Cerisola G., Rodrigo M. A., Panizza M. (2018). Removal of Procion Red MX-5B Dye
from Dastewater by Donductive-Diamond Electrochemical Oxidation. Electrochim. Acta.

[27] Mao X., Hong S., Zhu H., Lin H., Wei L., Gan F. (2008). Alternating pulse current in electrocoagulation
for wastewater treatment
to prevent the passivation of al electrode. J. Wuhan Univ. Technol., Mater. Sci. Ed.

[28] Núñez J, Yeber M., Cisternas N., Thibaut R., Medina P., Carrasco C. (2019). Application of Electrocoagulation
for the Efficient
Pollutants Removal to Reuse the Treated Wastewater in the Dyeing Process
of the Textile Industry. J. Hazard. Mater.

[29] Aitbara A., Khelalfa A., Bendaia M., Abrane R., Amrane A., Hazourli S. (2021). Treatment of Dairy
Wastewater by Electrocoagulation
Using A-U4G (2017-Al) Alloy and Pure Aluminum as Electrode Material. Euro-Mediterr. J. Environ. Integr..

[30] García-Orozco V. M., Linares-Hernández I., Natividad R., Balderas-Hernández P., Alanis-Ramírez C., Barrera-Díaz C. E., Roa-Morales G. (2022). Solar-Photovoltaic
Electrocoagulation of Wastewater from a Chocolate Manufacturing Industry:
Anodic material Effect (Aluminium. Copper and Zinc) and Life Cycle
Assessment. J. Environ. Chem. Eng.

[31] Othmani A., Kadierb A., Singhd R., Igwegbee C. A., Bouzidf M., Aquatarg M. O., Khandayi W. A., Botej M. E., Damirik F., Gökkus O., Sher F. (2022). A Comprehensive Review on Green Perspectives
of Electrocoagulation Integrated with Advanced Processes for Effective
Pollutants Removal from Water Environment. Environ.
Res..

[32] Zaidi S., Chaabane T., Sivasankar V., Darchen A., Maachi R., Msagati T. A. M. (2019). Electro-Coagulation
Coupled Electro-Flotation Process:
Feasible Choice in Doxycycline Removal from Pharmaceutical Effluents. Arab. J. Chem..

[33] Brandi J., Wilson-Wilde L. (2013). Standard Methods. Encycl. Forensic
Sci..

[34] Sampaio S. C., Silvestro M. G., Frigo E. P., Borges C. M. (2007). Relação
Entre Série de Sólidos e Condutividade Elétrica
em Diferentes Águas Residuárias. Irriga.

[35] Rusydi, A. F. Correlation Eetween Conductivity and Total Dissolved Solid in Various Type of Water: A Review. In IOP conference series: earth and environmental science; IOP Publishing Ltd.: Indonesia, 2018. DOI: 118, 012019. DOI: 10.1088/1755-1315/118/1/012019.

[36] Asaithambi P., Beyene D., Aziz A. R. A., Alemayehu E. (2018). Removal of
Pollutants with Determination of Power Consumption from Landfill Leachate
Wastewater Using an Electrocoagulation Process: Optimization Using
Response Surface Methodology (RSM). Appl. Water
Sci..

[37] Ebba M., Asaithambi P., Alemayehu E. (2022). Development of Electrocoagulation
Process for Wastewater Treatment: Optimization by Response Surface
Methodology. Heliyon.

[38] Driscoll W. C. (1996). Robustness
of the ANOVA and Tukey-Kramer Statistical Tests. Comput. Ind. Eng..

[39] Ghaedi M., Shojaeipour E., Ghaedi A. M., Sahraei R. (2015). Isotherm and kinetics
study of malachite green adsorption onto copper nanowires loaded on
activated carbon: Artificial Neural Network Nodeling and Genetic Algorithm
Optimization. Spectrochim. Acta A Mol. Biomol.
Spectrosc..

[40] Rangseesuriyachai T., Pinpatthanapong K., Boonnorat J., Jitpinit S., Pinpatthanapong T., Mueansichai T. (2024). Optimization of COD and TDS Removal from High-Strength
Hospital Wastewater by Electrocoagulation Using Aluminium and Iron
Electrodes: Insights from Central Composite Rotational Design. J. Environ. Chem. Eng..

[41] Ahmed A. W., Atiya M. A., M-Ridha M. J. (2023). Treatment
of Dairy Wastewater by
Electrocoagulation Using Iron Filings Electrodes. Baghdad Sci. J..

[42] Amani-Ghadim A. R., Abera S., Oladc A., Ashassi-Sorkhabi H. (2013). Optimization
of Electrocoagulation Process for Removal of an Azo Dye Using Response
Surface Methodology and Investigation on the Occurrence of Destructive
side Reactions. Chem. Eng. Process..

[43] Maroneze M. M., Zepka L. Q., Vieira J. G., Queiroz M. I., Jacob-Lopes E. A (2014). tecnologia
de Remoção de Fósforo: Gerenciamento do Elemento
em Resíduos Industriais. Rev. Ambient.
Água..

[44] Dassey A., Theegala C. (2012). Optimizing the Air Dissolution Parameters in an Unpacked
Dissolved Air Flotation System. Water.

[45] Muñoz-Alegría J. A., Muñoz-España E., Flórez-Marulanda J. F. (2021). Dissolved
Air Flotation: A Review from the Perspective of System Parameters
and Uses in Wastewater Treatment. TecnoLógicas.

[46] Rubio J., Souza M. L., Smith R. W. (2002). Overview
of Flotation as a Wastewater
Treatment Technique. Miner. Eng..

[47] Elabbas S., Ouazzani N., Mandi L., Berrekhis F., Perdicakis M., Pontvianne S., Pons M. N., Lapicque F., Leclerc J. P. (2016). Treatment of Highly
Concentrated Tannery Wastewater
Using Electrocoagulation: Influence of The Quality of Aluminium Used
for the Electrode. J. Hazard. Mater..

[48] Jang G. G., Keum J. K., Dutta S., Damron J. T., Wiechert A. I., Halbert C. E., Browning J. F., Hensley D. K., Jassby D., Hatzell M. C., Tsouris C. (2025). Understanding
the Dissolution and
Passivation of an Aluminum Electrode during Electrocoagulation of
Groundwater Using Neutron and X-ray Reflectometry. ACS Appl. Mater. Interfaces..

[49] Zini L. P., Longhi M., Jonko E., Giovanela M. (2020). Treatment
of Automotive Industry Wastewater by Electrocoagulation Using Commercial
Aluminum Electrodes. Process Saf. Environ. Prot..

[50] Devlin T. R., Kowalski M. S., Pagaduan E., Zhang X., Wei V., Oleszkiewicz J. A. (2019). Electrocoagulation
of Wastewater Using Aluminum, Iron,
and Magnesium Electrodes. J. Hazard. Mater..

[51] Merzouk B., Madani K., Sekki A. (2010). Using Electrocoagulation-Electroflotation
Technology to Treat Synthetic Solution and Textile Wastewater. two
Case Studies. Desalination..

[52] Yasri N., Nightingale M., Cleland K. J., Roberts E. P. (2022). The Impact of a
Magnetic Field on Electrode Fouling During Electrocoagulation. Chemosphere.

[53] Ye Z., Steter J. R., Centellas F., Cabot P. L., Brillas E., Sirés I. (2019). Photoelectro-Fenton
as Post-Treatment for Electrocoagulated
Benzophenone-3-loaded Synthetic and Urban Wastewater. J. Clean. Prod..

[54] Duan J., Wang J., Graham N., Wilson F. (2002). Coagulation
of Humic
Acid by Aluminium Sulphate in Saline Water Conditions. Desalination.

[55] Brasil. Resolução n° 430, de 13 de maio de 2011. Dispõe Sobre Condições e Padrões para Lançamento de Efluentes. DOU: Brasília, 2011.

[56] Demirhan E. (2020). Response Surface
Methodology Approach for Adsorptive Removal of Reactive Blue 19 Onto
Green Pea Pod. Water Sci. Technol..

[57] Guest J. S., Skerlos S. J., Barnard J. L., Beck M. B., Daigger G. T., Hilger H., Jackson S. J., Karvazy K., Kelly L., Macpherson L., Mihelcic J. R., Pramanik A., Raskin L., Van Loosdrecht M. C. M., Yeh D., Love N. G. (2009). A New Planning and
Design Paradigm to Achieve Sustainable Resource Recovery from Wastewater. Environ. Sci. Technol..

[58] Rekha H. B., Murthy U. N. (2016). Decolorization of Reactive dye Solutions by Electrocoagulation
Using Iron Electrodes. Nat. Environ. Pollut.
Technol..

[59] Das P. P., Sharma M., Purkait M. K. (2022). Recent Progress on Electrocoagulation
Process for Wastewater Treatment: A Review. Sep. Purif. Technol..

[60] Rainert K. T., Nunes H. C. A., Gonçalves M. J., Helm C. V., Tavares L. B. B. (2021). Decolorization
of the Synthetic Dye Remazol Brilliant Blue Reactive (RBBR) by *Ganoderma lucidum* on Bio-Adsorbent of the Solid Bleached
Sulfate Paperboard Coated with Polyethylene Terephthalate. J. Environ. Chem. Eng..

[61] Aljaberi F. Y. (2018). Studies
of Autocatalytic Electrocoagulation Reactor for Lead Removal from
Simulated Wastewater. J. Environ. Chem. Eng..

[62] Naje A. S., Chelliapan S., Zakaria Z., Ajeel M. A., Alaba P. A. (2017). A Review
of Electrocoagulation Technology for the Treatment of Textile Wastewater. Rev. Chem. Eng..

[63] Chen P., Li J., Xie N. (2023). Study on Influencing
Parameters of Total Phosphorus
Degradation in Cattle Farm Wastewater by Electrocoagulation Using
Magnesium, Aluminum, and Iron Electrodes. Water.

[64] Medvidović N. V., Vrsalović L., Svilović S., Bilušić A., Jozić D. (2023). Electrocoagulation Treatment of Compost Leachate Using
Aluminium Alloy, Carbon Steel, and Zinc Anode. Appl. Surf. Sci. Adv..

[65] Rahul K. B., Bhuvaneshwari S., Majeed F., Aravind S. P. (2022). Development
and
Applicability of Aluminium-Copper Alloy Electrodes for Dairy Wastewater
Treatment. J. Water Process Eng..

[66] Liu Y. H., Bootwala Y. Z., Jang G. G., Keum J. K., Khor C. M., Hoek E. M. V., Jassby D., Tsouris C., Mothersbaugh J., Hatzell M. C. (2022). Electroprecipitation Mechanism Enabling
Silica and
Hardness Removal through Aluminum-Based Electrocoagulation. ACS ES&T Eng..

[67] Oliveira, J. T. Estudo de Eficiência de Eletrodos com e sem Filmes Depositados por Nitretação Aplicados ao Tratamento de Efluentes Têxteis Utilizando a Tecnologia de Eletrocoagulação; Dissertação de Mestrado, Universidade Federal do Ceará: Fortaleza, Brasil, 2017.

[68] Agência de Notícias da Indústria. Custo da Energia Elétrica para Indústria. 2021. https://noticias.portaldaindustria.com.br/noticias/inovacao-e-tecnologia/custo-da-energia-eletrica-para-industria (accessed Jan 26, 2023).

[69] Ankoliya D., Mudgal A., Sinha M. K., Patel V., Patel J. (2023). Application
of Electrocoagulation Process for the Treatment of Dairy Wastewater:
A Mini Review. Mater. Today Proc..

[70] Sangal V. K., Mishra I. M., Kushwaha J. P. (2013). Electrocoagulation
of Soluble Oil
Wastewater: Parametric and Kinetic Study. Sep.
Sci. Technol..

[71] L̷uba M., Mikol̷ajczyk T., Pierozyński B., Smoczyński L., Wojtacha P., Kuczyński M. (2020). Electrochemical
Degradation of Industrial
Dyes in Wastewater through the Dissolution of Aluminum Sacrificial
Anode of Cu/Al Macro-Corrosion Galvanic Cell. Molecules.

[72] Brasil. Resolução n° 357, de 17 de março de 2005; Ministério do Meio Ambiente: Brasília, 2005.

[73] Brasil. Portaria GM/MS n° 888, de 4 de maio de 2021; Dispõe sobre os padrões de potabilidade da água para consumo humano; Ministério da Saúde: Brasília, 2021.

[74] Souza, S. P. M. C. Potencial do Tratamento Eletroquímico Oxidativo Associado à Adsorção para Remediação de Corantes Têxteis; Tese de Doutorado, Universidade Federal do Rio Grande do Norte: Natal, Brasil, 2015.

[75] Kloprogge J. T., Ruan H. D., Frost R. L. (2002). Thermal Decomposition of Bauxite
Minerals: Infrared Emission Spectroscopy of Gibbsite, Boehmite, and
Diaspore. J. Mater. Sci..

[76] González-Gómez M. A., Belderbos S., Yañez-Vilar S., Piñeiro Y., Cleeren F., Bormans G., Deroose C. M., Gsell W., Himmelreich U., Rivas J. (2019). Development of Superparamagnetic
Nanoparticles Coated with Polyacrylic Acid and Aluminum Hydroxide
as an Efficient Contrast Agent for Multimodal Imaging. Nanomaterials.

[77] Saniger J. M. (1995). Al–O
Infrared Vibrational Frequencies of γ-Alumina. Mater. Lett..

[78] Djordjević C. (1961). Metal-Oxygen
Vibration Modes in the Infrared Spectra of Aluminium, Gallium, and
Indium Tris-Acetylacetonates. Spectrochim. Acta.

[79] Rajaniemi K., Tuomikoski S., Lassi U. (2021). Electrocoagulation Sludge Valorization
– A Review. Resources.

