# Supplementary material for: Optimizing Electrocoagulation for Textile Effluent Treatment: Operational Efficiency and Environmental Assessment of Remazol Red Dye Removal
Source: ACS Omega. 2025 Dec 12;10(51):62962–78. doi: 10.1021/acsomega.5c08815 (PMC12756720; doi:10.1021/acsomega.5c08815)
Supplement: Supplementary file 1 [file ao5c08815_si_001.pdf]

Optimizing Electrocoagulation for Textile Effluent Treatment: Operational Efficiency and Environmental Assessment of Remazol Red Dye Removal

Francisco F. S. Cruz[a], Mauro C. C. Góes[b], Claudemir G. Santana.[c], Tiago G. Santos[a], Mauricio Boscolo[d], Rita C. S. Luz[a] and Cícero W. B. Bezerra[a,]*

[a] *Departamento de Química, Universidade Federal do Maranhão, Av. dos Portugueses, 1966, Bacanga - CEP 65080-805, São Luís – MA, Brazil*
[b] *Secretaria de Educação do Estado do Maranhão, Unidade Regional de Educação de São Luís, Rua do Cema, 39 - Vila Palmeira - CEP 65047-400, São Luís – MA, Brazil*
[c] *Departamento de Tecnologia Química, Universidade Federal do Maranhão, Av. dos Portugueses, 1966, Bacanga - CEP 65080-805, São Luís – MA, Brazil*
[d] *Instituto de Biociências, Letras e Ciências Exatas – IBILCE/UNESP Campus de São Jose do Rio Preto –SP, Brasil, CEP 15054-000, Brazil*

This supplementary material includes detailed statistical analyses, additional figures, and raw data tables to support the findings presented in the main manuscript.

1. Statistical Analysis

1.1. Exclusion of the Pressure Variable in Subsequent Experiments

Two statistical studies were conducted to evaluate the effect of pressure on dye removal results: the first considered only the central points, which shared the same experimental conditions, while the second analyzed all the points from the factorial design using Tukey's test.

The results in Table S1 illustrate the impact of pressure on dye removal percentage under central point conditions, comparing scenarios both in the absence and presence of pressure (4 atm).

Table S1: Percentage removal (%Rr) of remazol red dye under central point conditions and with and without the influence of DAF coupling (4 atm of pressure).

**Central Point**	**% Rr**	
	**EC**	**EC/DAF**
**19**	96.8	95.0
**21**	96.9	97.2
**18**	96.6	96.5
**17**	97.4	97.3
**20**	95.6	97.4
**Mean (Md)**	96.66	96.68
**SD**	0.5919	0.8975

In order to evaluate the statistical significance of the observed differences between the two sets of conditions, we assumed the null hypothesis that there is no significant difference between the dye removal percentages with and without DAF coupling. A paired t-test was applied to compare the means of the two samples. The critical t-value for $P = 0.05$, with 4 degrees of freedom, is 2.78. The experimental $|t|$ value (0.055) was found to be less than the critical t-value (2.78), thereby confirming the null hypothesis.

This statistical result indicates that there are no significant differences between the experiments conducted with and without pressure under central point conditions. The system demonstrated reproducibility, suggesting that the addition of pressure did not have a significant impact on the efficiency of dye removal in this context. Consequently, it was decided not to employ the DAF operation in subsequent trials, as it did not provide any measurable improvement under the tested conditions. The decision to exclude the DAF process from further experiments highlights the efficiency of the EC process alone in achieving substantial dye removal percentages, which remain consistently high across trials. It suggests that, at least under the tested conditions, the electrocoagulation process can achieve similar outcomes without the need for additional pressure, thus simplifying the operational complexity and potentially reducing costs.

Figure S1 presents the four groups formed for the Tukey test. In general, removal rates were similar. Removal indices ranged from 94.5% to 97.4% without dissolved air pressure, and from 89.5% to 97.3% with dissolved air pressure.

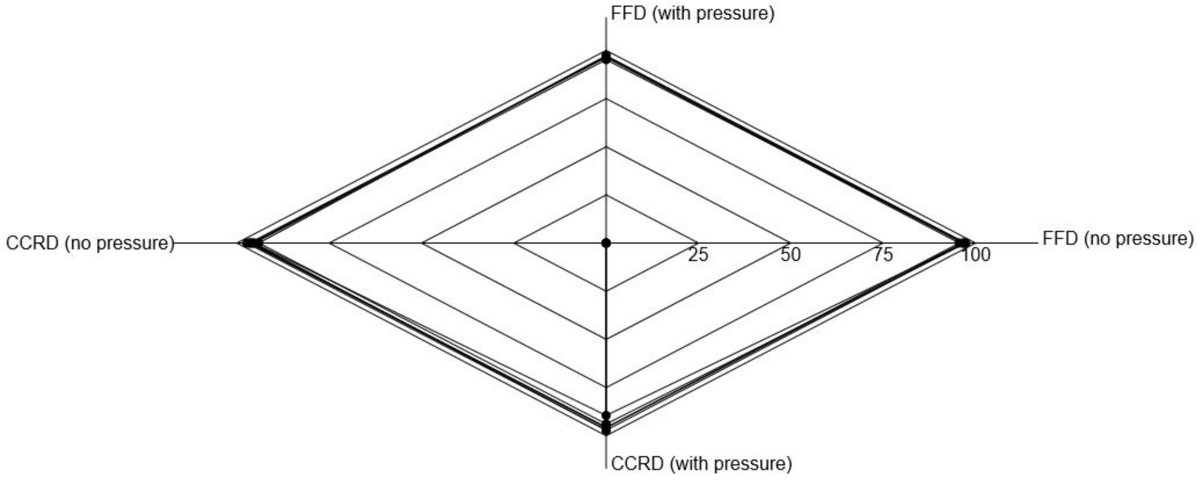

Figure S1: Removal rate of remazol red after each experiment for the central points. FFD - fractional factorial design. CCRD - central composite rotational design.

To identify significant differences among the obtained values, an analysis of variance (ANOVA) was performed for the removal rates in the experiments related to the central points. Table S2 shows that p-value > 0.05 and Fcal < Fcritical.

Table S2: ANOVA (Tukey's test 95%) for significant comparisons in electrocoagulation experiments related to central points.

Source of variation	SS [a]	DF [b]	MS [c]	F_{ratio}	$F_{critical}$	P_{value}
**Between groups**	10.619	3	3.539	1.331	3.100	0.292
**Within groups**	53.166	20	2.658			
**Total**	63.785	23	0.2731			

[a] Sum square, [b] degrees of freedom, [c] mean square, [d] intraclass correlation coefficient, [e] effect size

Thus, from a statistical standpoint, the removal of remazol red showed no significant differences between the formed groups, indicating that the pressure factor does not justify the high removal rate. Table S3 presents the possible comparisons among the groups represented in Figure S1.

Table S3: Tukey test results for non-significant comparisons (p-value > 0.05) between trials related to central points.

Comparisons	Mean difference	p-value
**FFD no pressure and FFD with pressure**	0.15	0.999
**FFD no pressure and CCRD no pressure**	0.77	0.854
**FFD no pressure and CCRD with pressure**	1.77	0.324
**FFD with pressure and CCRD no pressure**	0.62	0.916
**FFD with pressure and CCRD with pressure**	1.53	0.400
**CCRD no pressure and CCRD with pressure**	0.91	0.725

Table S4. Variables evaluated by central rotational composite design.

Essay	[a] PD v	[b] EP u	[c] ES cm	[d] T min	[e] pH_i -	[f] Ci mgL^{-1}	[g] Sal g L^{-1}	[h] **Λ** mscm^{-1}	[i] TDS ppm	[j] pH_f -	[k] Col uH	[l] Tur NTU	[m] Rr %
1	5	1	1	30	2	10	0.78	1.60	1.02	7.23	2.00	0.69	75.00
2	5	1	1	30	12	150	1.20	2.39	1.53	10.03	96.2	0.40	40.18
3	5	1	1	120	5	150	1.26	2.50	1.60	6.88	25.4	1.10	23.33
4	5	1	1	120	12	10	0.94	1.90	1.22	9.35	3.85	0.80	48.90
5	5	1	3	30	5	150	1.53	2.98	1.91	6.38	762	3.50	44.11
6	5	1	3	30	12	10	1.18	2.34	1.50	9.95	54.7	0.83	11.10
7	5	1	3	120	5	10	1.68	3.25	2.08	8.45	39.6	0.19	66.50
8	5	1	3	120	12	150	0.95	1.92	1.23	10.13	86.5	0.88	6.63
9	5	3	1	30	5	150	1.19	2.36	1.51	6.50	23.1	0.71	78.51
10	5	3	1	30	12	10	1.42	2.79	1.79	9.42	3.85	0.63	57.60
11	5	3	1	120	5	10	0.69	1.44	0.92	9.50	8.34	1.51	85.40
12	5	3	1	120	12	150	1.17	2.33	1.49	11.50	19.6	61.00	49.57
13	5	3	3	30	5	10	1.00	2.01	1.29	8.67	5.00	0.30	76.50
14	5	3	3	30	12	150	1.45	2.83	1.81	11.56	91.3	0.56	36.48
15	5	3	3	120	5	150	0.98	1.97	1.26	9.30	23.1	0.87	75.33
16	5	3	3	120	12	10	0.94	1.90	1.22	9.75	1.0	1.73	49.80
17	20	1	1	30	5	150	0.77	1.58	1.01	6.85	30.8	0.65	83.96
18	20	1	1	30	12	10	1.25	2.48	1.59	9.53	38.5	0.73	12.70
19	20	1	1	120	5	10	0.93	1.89	1.21	8.95	0.70	1.10	92.27
20	20	1	1	120	15	150	1.25	2.48	1.59	9.50	89.3	2.60	12.66
21	20	1	3	30	5	10	0.94	1.91	1.22	8.68	2.00	1.90	33.20
22	20	1	3	30	12	150	1.30	2.57	1.64	8.51	31.2	0.48	56.67
23	20	1	3	120	5	150	1.18	2.34	1.50	9.45	30.8	0.69	80.44
24	20	1	3	120	12	10	1.25	2.48	1.59	9.24	1.97	0.74	42.60
25	20	3	1	30	5	10	0.80	1.64	1.05	8.32	0	0.31	100.0
26	20	3	1	30	12	150	1.16	2.31	1.48	9.70	20.1	0.40	51.48

Essay	[a] PD v	[b] EP u	[c] ES cm	[d] T min	[e] pH_i -	[f] Ci mg L^{-1}	[g] Sal g L^{-1}	[h] **Λ** mscm^{-1}	[i] TDS ppm	[j] pH_f -	[k] Col uH	[l] Tur NTU	[m] Rr %
27	20	3	1	120	5	150	1.43	2.80	1.79	9.84	7.40	0.80	98.84
28	20	3	1	120	12	10	0.74	1.53	0.98	9.90	1.00	0.03	91.70
29	20	3	3	30	5	150	0.95	1.92	1.23	9.36	21.54	1.50	45.16
30	20	3	3	30	12	10	0.81	1.67	1.07	9.05	3.83	1.02	69.60
31	20	3	3	120	5	10	0.92	1.86	1.19	9.89	0	0.40	98.00
32	20	3	3	120	12	150	1.01	2.04	1.31	9.55	7.09	0.12	98.67
33	5	2	2	75	8.5	80	0.95	1.93	1.24	9.23	7.41	1.04	75.31
34	30.34	2	2	75	8.5	80	1.42	2.79	1.79	9.42	1.00	0.63	97.16
35	12.5	1	2	75	8.5	80	1.11	2.21	1.41	8.95	7.93	2.20	40.00
36	12.5	4	2	75	8.5	80	1.35	2.65	1.70	9.56	3.85	0.89	90.22
37	12.5	2	1	75	8.5	80	1.08	2.17	1.39	9.24	3.50	1.12	93.98
38	12.5	2	4	75	8.5	80	1.27	2.52	1.61	9.02	1.93	2.50	92.40
39	12.5	2	2	30	8.5	80	1.11	2.21	1.41	9.50	5.46	0.21	95.26
40	12.5	2	2	182	8.5	80	0.51	1.08	0.69	8.89	0	0.97	98.36
41	12.5	2	2	75	0.18	80	1.29	2.54	1.63	6.95	3.71	0.68	96.80
42	12.5	2	2	75	13.32	80	1.30	2.56	1.64	10.65	3.85	0.50	61.28
43	12.5	2	2	75	8.5	10	1.14	2.28	1.46	7.85	0	0.61	93.90
44	12.5	2	2	75	8.5	246.5	2.13	4.04	2.59	10.23	0.20	1.03	87.36
45 (C)	12.5	2	2	75	8.5	80	0.84	1.72	1.10	9.02	3.92	0.20	96.07
46 (C)	12.5	2	2	75	8.5	80	1.10	2.2	1.41	9.01	5.55	0.84	95.51
47 (C)	12.5	2	2	75	8.5	80	0.91	1.85	1.18	8.80	3.10	0.33	94.05
48 (C)	12.5	2	2	75	8.5	80	0.85	1.73	1.11	9.13	4.77	0.26	97.30
49 (C)	12.5	2	0	75	8.5	80	0.53	1.12	0.72	9.30	3.68	0.50	96.40
50 (C)	12.5	2	2	75	8.5	80	0.78	1.61	1.03	8.45	4.46	0.44	95.32
51 (C)	12.5	2	2	75	8.5	80	1.16	2.31	1.48	8.85	5.00	0.37	96.60

[a] Potential difference, [b] electrode pairs, [c] electrode space, [d] time, [e] initial pH, [f] initial concentration, [g] salinity, [h] conductivity, [i] total dissolved solids, [j] final pH, [k] color, [l] turbidity, [m] removal rate.

1.2. Anova DCCR Data

Table S5. Analysis of variance (ANOVA) for salinity, final conductivity, total solids, final pH, color, turbidity and removal.

Variable	Salinity $R^2 = 0.6123$		Final conductivity $R^2 = 0.6115$		Total dissolved solids $R^2 = 0.6114$		Final pH $R^2 = 0.8310$	
Factor	[a] SS	P-value	[a] SS	P-value	[a] SS	P-value	[a] SS	P-value
[b] PD (L)	0.007039	0.702340	0.02148	0.722756	0.008928	0.720487	0.10891	0.271025
PD (Q)	0.063523	0.273734	0.23292	0.266619	0.098457	0.259341	0.22024	0.135522
[c] EP (L)	0.031023	0.432179	0.10807	0.436147	0.042719	0.443309	4.62316	0.000219
EP (Q)	0.097192	0.186821	0.33121	0.194500	0.134588	0.195553	0.14168	0.216065
[d] ES (L)	0.054889	0.305653	0.18132	0.321432	0.074243	0.320952	0.44631	0.049534
ES (Q)	0.057009	0.297310	0.21410	0.284845	0.086361	0.287640	0.04432	0.468737
[e] T (L)	0.077914	0.230595	0.27283	0.233278	0.109968	0.236084	2.30374	0.001413
T (Q)	0.060327	0.284933	0.21738	0.281532	0.091998	0.273906	0.08808	0.317511
[f] pH_i (L)	0.023728	0.489385	0.08847	0.478970	0.037460	0.471444	22.50319	0.000002
pH_i (Q)	0.158416	0.105846	0.53987	0.111541	0.223756	0.109490	0.05172	0.435545
[g] Ci (L)	0.546354	0.012329	1.79440	0.014513	0.733778	0.014487	0.53651	0.036035
Ci (Q)	0.721177	0.006661	2.38479	0.007816	0.981265	0.007692	0.00830	0.749349
PD-EP (L)	0.004278	0.765167	0.01575	0.760918	0.006328	0.762859	0.26281	0.108706
PD-ES (L)	0.033153	0.417641	0.10238	0.447858	0.042778	0.443009	0.21780	0.137329
PD-T (L)	0.109278	0.165215	0.36765	0.174833	0.152628	0.171912	0.04500	0.465496
PD-pH_i (L)	0.015753	0.570552	0.05528	0.572592	0.022578	0.572649	7.16311	0.000064
PD-Ci (L)	0.003003	0.802154	0.00878	0.819996	0.003828	0.814167	0.01805	0.639222
EP-ES (L)	0.147153	0.116449	0.49253	0.125242	0.200028	0.126171	0.00001	0.990060
EP-T (L)	0.060378	0.284750	0.20640	0.292844	0.087153	0.285649	0.10811	0.272603
EP-pH_i (L)	0.007503	0.693281	0.02475	0.703555	0.010153	0.703008	0.58320	0.030960
EP-Ci (L)	0.073153	0.243683	0.24675	0.254307	0.100128	0.255761	1.30411	0.005721
ES-T (L)	0.000253	0.941862	0.00113	0.934854	0.000378	0.940980	0.56180	0.033158
ES-pH_i (L)	0.077028	0.232951	0.26100	0.242485	0.109278	0.237390	1.66531	0.003192
ES-Ci (L)	0.048828	0.331592	0.16965	0.336224	0.069378	0.336023	0.12005	0.250238
T-pH_i (L)	0.216153	0.068013	0.73508	0.072509	0.298378	0.073221	2.58781	0.001045
T-Ci (L)	0.001653	0.852340	0.00428	0.873634	0.001653	0.877073	0.29645	0.092446
pH_i-Ci(L)	0.010878	0.635899	0.03578	0.648253	0.015753	0.636045	2.77301	0.000872
lack of fit	1.375735	0.230000	4.59679	0.254506	1.891689	0.251474	9.46384	0.009904
pure error	0.262686		0.93189		0.380743		0.44474	
Total SS	4.226051		14.23070		5.848592		58.64086	

[a] Sum square, [b] potential difference, [c] electrode pairs, [d] electrode space, [e] time, [f] initial pH, [g] initial concentration.

Continuation table S5

**Variable**	**Color** $R^2 = 0.6362$		**Tubidity** $R^2 = 0.6323$		**Removal** $R^2 = 0.7195$	
Factor	[a] SS	P-value	[a] SS	P-value	[a] SS	P-value
[b] PD (L)	1923.95	0.000000	92.232	0.000000	2008.88	0.000000
PD (Q)	560.17	0.000002	5.731	0.000028	1508.85	0.000000
[c] EP (L)	3387.26	0.000000	56.290	0.000000	7030.59	0.000000
EP (Q)	671.02	0.000000	7.539	0.000013	4472.71	0.000000
[d] ES (L)	240.09	0.000000	68.494	0.000000	305.69	0.000003
ES (Q)	470.48	0.000000	13.601	0.000002	876.99	0.000000
[e] T (L)	649.11	0.000000	88.055	0.000000	560.15	0.000001
T (Q)	471.34	0.000000	4.280	0.000065	615.54	0.000000
[f] pH_i (L)	1495.18	0.000001	73.185	0.000000	5880.66	0.000000
pH_i (Q)	533.79	0.000082	4.280	0.000065	2341.80	0.000000
[g] Ci (L)	6088.34	0.000000	95.600	0.000000	481.44	0.000001
Ci (Q)	331.98	0.000000	5.636	0.000030	1089.75	0.000000
PD-EP (L)	62.75	0.000026	124.939	0.000000	64.72	0.000261
PD-ES (L)	2527.43	0.000000	105.888	0.000000	165.48	0.000018
PD-T (L)	571.98	0.000427	116.167	0.000000	972.74	0.000000
PD-pH_i (L)	93.13	0.000058	109.483	0.000000	25.61	0.002967
PD-Ci (L)	550.87	0.000000	117.543	0.000000	341.06	0.000002
EP-ES (L)	34.59	0.000000	112.613	0.000000	7.81	0.037683
EP-T (L)	70.95	0.000239	120.552	0.000000	417.39	0.000001
EP-pH_i (L)	574.01	0.000001	118.003	0.000000	409.91	0.000001
EP-Ci (L)	550.71	0.253527	100.501	0.000000	113.51	0.000054
ES-T (L)	42.71	0.000001	148.307	0.000000	629.33	0.000000
ES-pH_i (L)	282.80	0.000057	122.892	0.000000	486.95	0.000001
ES-Ci (L)	1.13	0.000000	113.892	0.000000	459.27	0.000001
T-pH_i (L)	338.33	0.000000	135.096	0.000000	11.22	0.018968
T-Ci (L)	71.01	0.000000	111.639	0.000000	532.60	0.000001
pH_i-Ci(L)	720.58	0.000002	99.793	0.000000	134.19	0.000033
lack of fit	12689.20	0.000000	1312.641	0.000000	11089.99	0.000000
pure error	4.25		0.268		6.64	
Total SS	34889.67		3570.727		39565.84	

[a] Sum square, [b] potential difference, [c] electrode pairs, [d] electrode space, [e] time, [f] initial pH, [g] initial concentration.

Table S6. Regression coefficients for salinity, final conductivity, total solids and final pH.

Variable	Salinity $R^2 = 0.6123$		Final conductivity $R^2 = 0.6115$		Total dissolved solids $R^2 = 0.6114$		Final pH $R^2 = 0.8310$	
Factor	[a] Regr. coeff.	P-value	[a] Regr. coeff.	P-value	[a] Regr. coeff.	P-value	[a] Regr. coeff.	P-value
Mean	0.891576	0.000029	1.809724	0.000019	1.158876	0.000018	8.940198	0.000000
[b] PD (L)	-0.012748	0.702340	-0.022269	0.722756	-0.014357	0.720487	0.050143	0.271025
PD (Q)	0.033573	0.273734	0.064288	0.266619	0.041798	0.259341	0.062514	0.135522
[c] EP (L)	-0.026762	0.432179	-0.049949	0.436147	-0.031405	0.443309	0.326706	0.000219
EP (Q)	0.041528	0.186821	0.076662	0.194500	0.048869	0.195553	0.050140	0.216065
[d] ES (L)	0.035598	0.305653	0.064701	0.321432	0.041401	0.320952	0.101509	0.049534
ES (Q)	0.031806	0.297310	0.061636	0.284845	0.039146	0.287640	0.028043	0.468737
[e] T (L)	-0.042413	0.230595	-0.079365	0.233278	-0.050387	0.236084	0.230624	0.001413
T (Q)	-0.032718	0.284933	-0.062107	0.281532	-0.040403	0.273906	0.039533	0.317511
[f] pH_i (L)	0.023406	0.489385	0.045195	0.478970	0.029408	0.471444	0.720791	0.000002
pH_i (Q)	0.053019	0.105846	0.097876	0.111541	0.063011	0.109490	-0.030293	0.435545
[g] Ci (L)	0.112312	0.012329	0.203539	0.014513	0.130158	0.014487	0.111296	0.036035
Ci (Q)	0.113123	0.006661	0.205709	0.007816	0.131954	0.007692	0.012133	0.749349
PD-EP (L)	-0.011562	0.765167	-0.022188	0.760918	-0.014062	0.762859	-0.090625	0.108706
PD-ES (L)	-0.032188	0.417641	-0.056562	0.447858	-0.036563	0.443009	-0.082500	0.137329
PD-T (L)	0.058437	0.165215	0.107188	0.174833	0.069063	0.171912	0.037500	0.465496
PD-pH_i (L)	0.022188	0.570552	0.041563	0.572592	0.026563	0.572649	-0.473125	0.000064
PD-Ci (L)	0.009687	0.802154	0.016562	0.819996	0.010938	0.814167	-0.023750	0.639222
EP-ES (L)	-0.067812	0.116449	-0.124062	0.125242	-0.079062	0.126171	-0.000625	0.990060
EP-T (L)	-0.043437	0.284750	-0.080312	0.292844	-0.052187	0.285649	0.058125	0.272603
EP-pH_i (L)	0.015312	0.693281	0.027813	0.703555	0.017813	0.703008	-0.135000	0.030960
EP-Ci (L)	0.047812	0.243683	0.087812	0.254307	0.055938	0.255761	0.201875	0.005721
ES-T (L)	-0.002813	0.941862	-0.005938	0.934854	-0.003437	0.940980	-0.132500	0.033158
ES-pH_i (L)	-0.049063	0.232951	-0.090312	0.242485	-0.058437	0.237390	-0.228125	0.003192
ES-Ci (L)	-0.039063	0.331592	-0.072812	0.336224	-0.046562	0.336023	0.061250	0.250238
T-pH_i (L)	-0.082187	0.068013	-0.151562	0.072509	-0.096563	0.073221	-0.284375	0.001045
T-Ci (L)	-0.007187	0.852340	-0.011563	0.873634	-0.007187	0.877073	0.096250	0.092446
pH_i-Ci(L)	-0.018438	0.635899	-0.033437	0.648253	-0.022188	0.636045	0.294375	0.000872

[a] Regression coefficient, [b] potential difference, [c] electrode pairs, [d] electrode space, [e] time, [f] initial pH, [g] initial concentration.

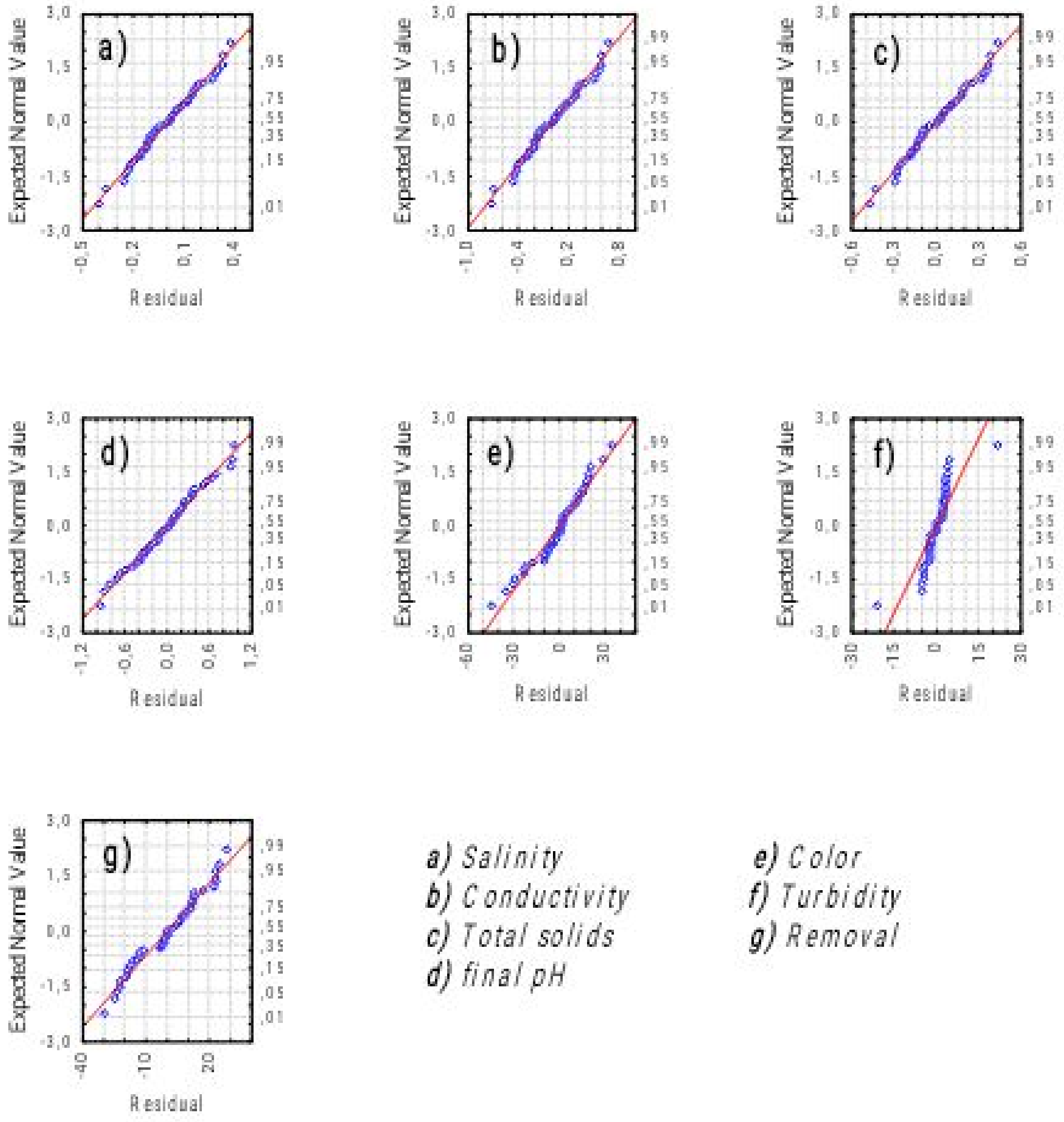

Figure S2. Normal probability plots of residuals for the experimental data obtained in the electrocoagulation process: (a) salinity, (b) conductivity, (c) total solids, (d) final pH, (e) color, (f) turbidity, and (g) removal

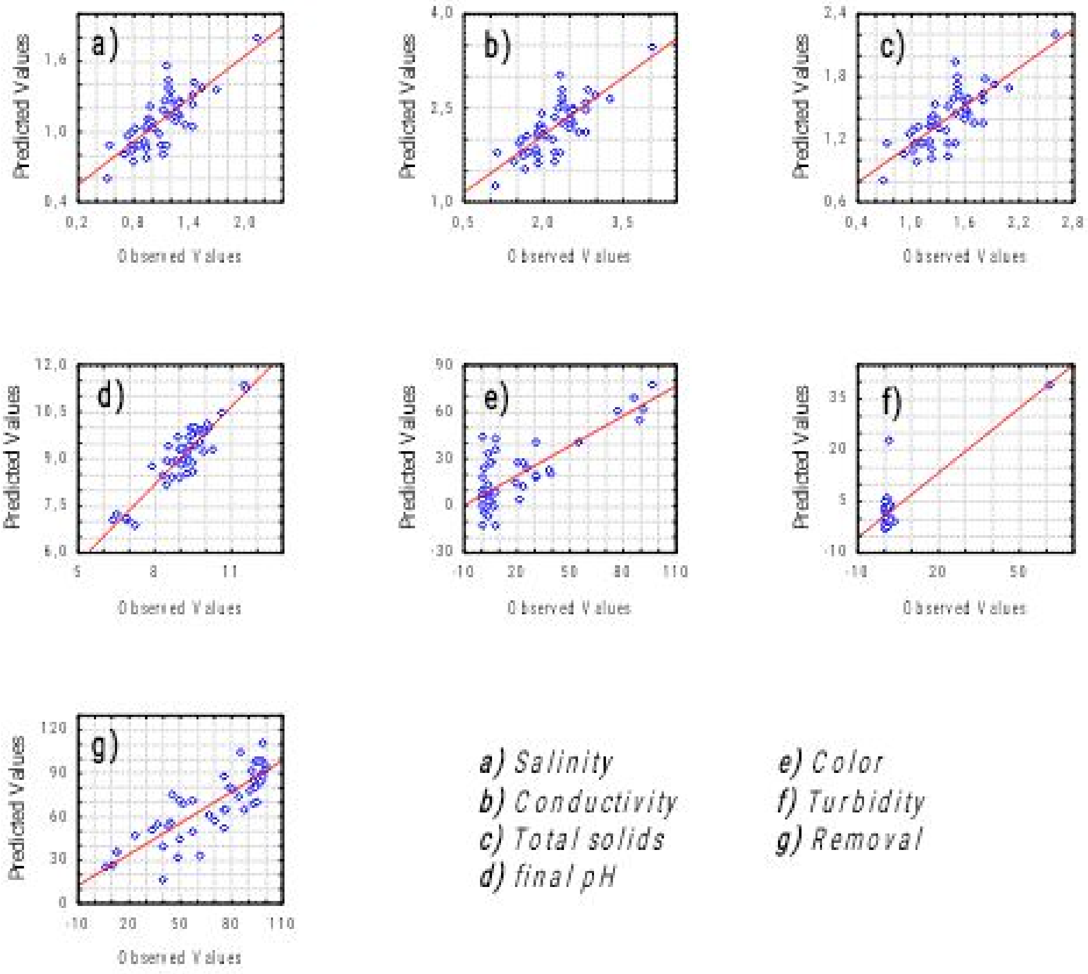

Figure S3. Plot of experimental vs predicted values by the mathematical models: (a) salinity, (b) conductivity, (c) total solids, (d) final pH, (e) color, (f) turbidity, and (g) removal efficiency

2. Electrode Consumption and Efficiency of the EC Process

Table S7: Mass Loss of Anodes and Energy Consumption under the Optimal Conditions Established by CCRD: V = 12 V; EP = 2; ES = 2 cm; t = 76 min; initial pH = 8.4; and Dye Concentration = 82 mg L⁻¹.

**Trial**	$\mathbf{m_{ia1} + m_{ia2\ (g)}}$	$\mathbf{m_{fa1} + m_{fa2\ (g)}}$	**□m** $_{(g)}$	**□m** $_{t\ (g)}$	**i** $_{(A)}$	$\mathbf{m_{ox\ (teo)}}$
**1**	12.334 + 12.169	12.159 + 11.874	0.175 + 0.295	0.470	0.923	0.392
**2**	12.825 + 12.482	12.619 + 12.176	0.206 + 0.306	0.512	0.976	0.415
**3**	11.671 + 12.304	11.529 + 12.051	0.142 + 0.253	0.395	0.879	0.374

Table S8: Energy Consumption and cost for electrocoagulation processes conducted under the optimal conditions established by CCRD (V = 12 V; EP = 2; ES = 2 cm; t = 76 min; initial pH = 8.4; and dye concentration = 82 mg L^{-1}).

Trial	i (A)	C_e (kWhm^{-3})	Cost (R$ m^{-3})	Cost (US$ m^{-3})
**1**	0.923	4.69	3.21	0.524
**2**	0.976	4.96	3.40	0.555
**3**	0.879	4.46	3.06	0,499

Analysis of Aluminum Electrode Composition, Effluents, and Generated Residues

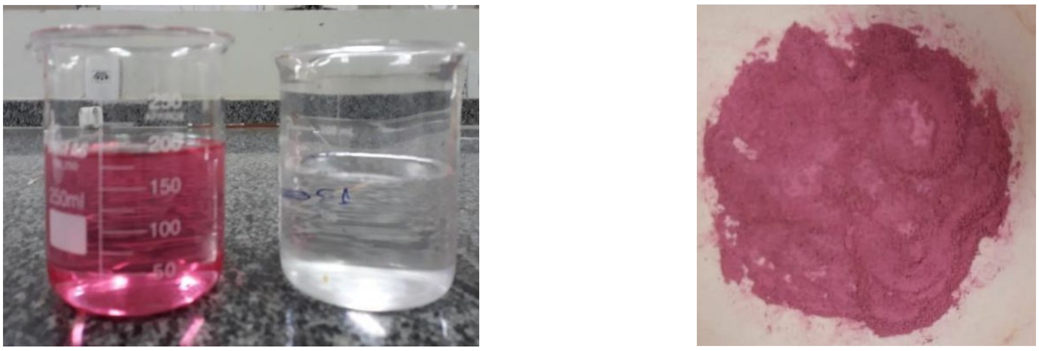

a) **b)**

Figure S4. (a) Visual appearance of the effluent before (concentration = 82 mg L^{-1}) and after treatment (voltage = 12 v, electrode pairs = 2, electrode spacing = 2 cm, time = 76 min, initial pH = 8.4); b) Sludge.

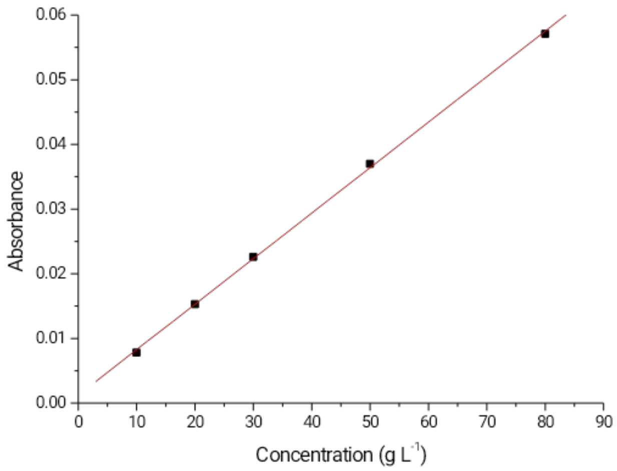

Figure S5: Analytical curve for aluminum determination by AAS. Equation: I = 0.00122 + 7.03766x10^{-4}; R = 0,99974.

Table S9: Aluminum content determined by AAS for samples: electrode, treated effluent, and produced sludge.

**Sample**	**Al content**
Electrode	99.80%
Treated effluent	0.92 ppm
Sludge	23.30 %

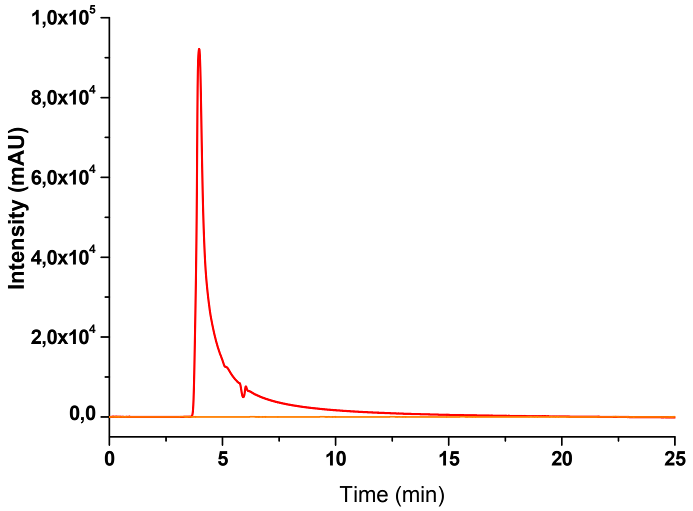

Figure S6. Chromatograms of synthetic effluent before (—) and after (—) EC Treatment. (Trial 7/DAF, table 3)

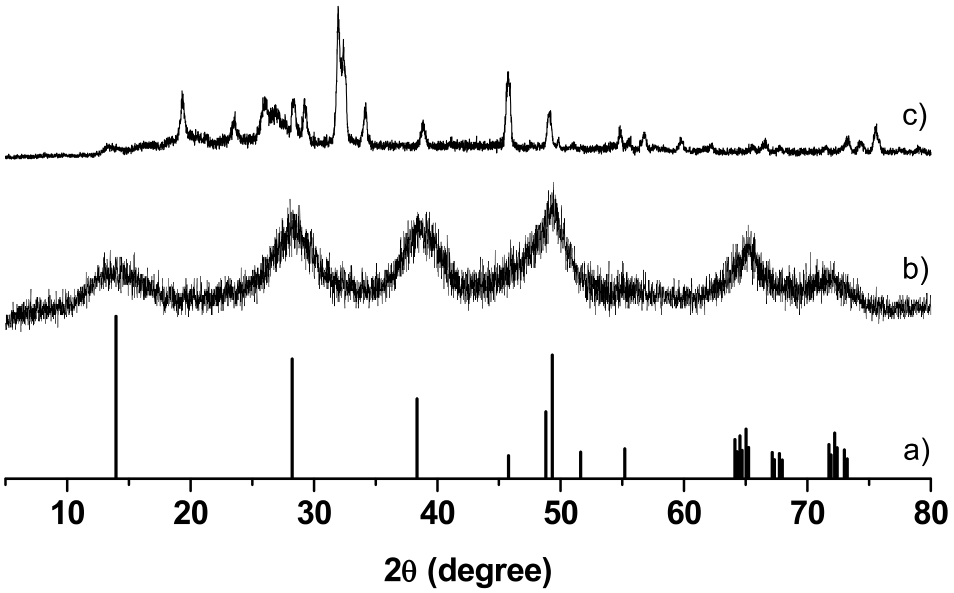

Figure S7: X-ray Diffraction Patterns: (a) Aluminum Oxyhydroxide Standard, (b) Sludge from the Electrocoagulation of Remazol Red, and (c) Remazol Red Dye.

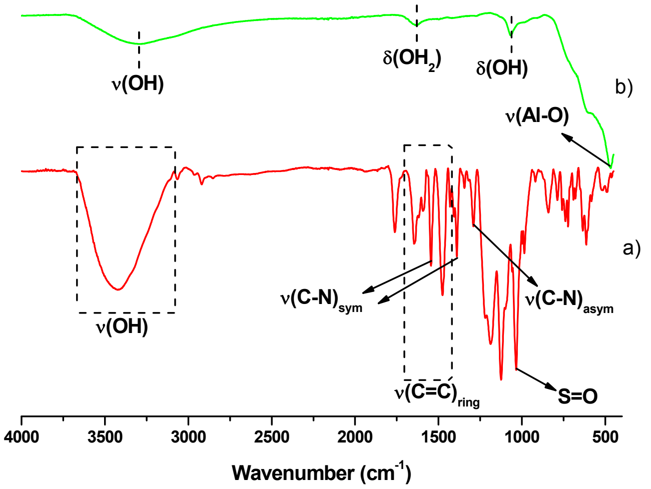

Figure S8. Vibrational Spectrum in the Infrared Region (FTIR) of a) Remazol Red Dye; b) sludge.

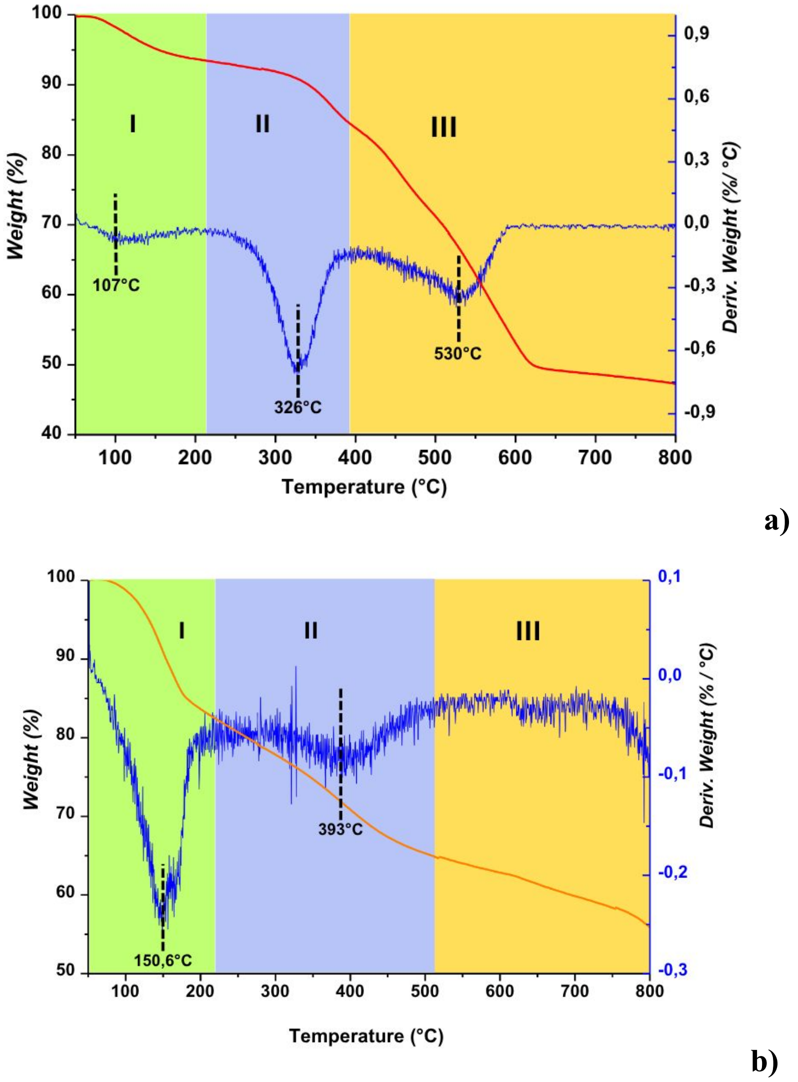

Figure S9. Thermogravimetric (TG and DTG) Curves of a) Remazol Red Dye; b) sludge, in a Synthetic Air Atmosphere (20 mL·min^{-1} Flow Rate)

Table S10: Thermal analysis parameters of the dye and the produced under synthetic air (20 mL min⁻¹).

Event	Sample	□T* (°C)	T_{onset*} (°C)	T_{max}* (°C)	Mass loss** (%)	DTG_{max*} (%·°C^{-1})	Assignment
I	Dye	50 – 205	50	107	6.3	–0.11	Dehydration (loss of adsorbed/bound water)
	Sludge	50 – 230	50	150.6	16.5	–0.24	Desorption of water and volatile organics
II	Dye	206 – 402	230	326	10.0	–0.69	Thermal degradation of dye's organic matrix
	Sludge	231 – 520	310	393	18.7	–0.09	Degradation of organic matter and AlOOH dehydroxylation
III	Dye	403 – 800	402	530	36.5	–0.34	Decomposition/carbonization
	Sludge	521 – 800	520	635	8.9	-0.05	Weak residual decomposition

*Temperatures obtained from the DTG curve.

**Values obtained from the TG curve.
